# Vitamin D Binding Protein, a Ligand of Integrin beta 1, Motivates Both Tumor Cells and Schwann Cells to Promote Perineural Invasion in Pancreatic Ductal Adenocarcinoma

**DOI:** 10.1002/advs.202511726

**Published:** 2025-09-09

**Authors:** Shan Zhang, Luju Jiang, Shuqi Cai, Zheqi Weng, Yuheng Zhu, Zhi‐Wei Cai, Hui Li, Qing Li, Li‐Peng Hu, Hong‐Fei Yao, Rong Hua, Yu Zhao, Dongxue Li, Xiao‐Mei Yang, Jun‐Feng Zhang, Shu‐Heng Jiang

**Affiliations:** ^1^ State Key Laboratory of Systems Medicine for Cancer Shanghai Cancer Institute Ren Ji Hospital School of Medicine Shanghai Jiao Tong University Shanghai 200240 P. R. China; ^2^ Department of General Surgery Hepato‐biliary‐pancreatic Center Huadong Hospital Fudan University Shanghai 200040 P. R. China; ^3^ Department of Biliary‐Pancreatic Surgery Ren Ji Hospital School of Medicine Shanghai Jiao Tong University Shanghai 200217 P. R. China; ^4^ Department of Bioengineering Imperial College London London SW7 2AZ UK; ^5^ Shanghai key laboratory for cancer systems regulation and clinical translation Department of General Surgery Jiading District Central Hospital Affiliated Shanghai University of Medicine & Health Sciences Shanghai 201800 P. R. China

**Keywords:** cancer neuroscience, neural invasion, pancreatic cancer, tumor microenvironment, VDBP

## Abstract

Perineural invasion (PNI) is a common pathological characteristic of pancreatic ductal adenocarcinoma (PDAC), closely linked to postoperative recurrence, metastasis, and unfavorable prognosis. Nevertheless, the precise mechanisms that govern PNI in PDAC remain poorly elucidated. Here, group‐specific component protein (GC) is identified as one of the most significantly upregulated genes related to PNI, primarily derived from malignant ductal cells compared to other cell types. GC knockdown attenuates PDAC cell invasiveness toward nerves, and this effect operates independently of vitamin D transport. Moreover, GC protein activates Schwann cells by inducing a dedifferentiation program, and enhances the mutual chemoattraction between PDAC cells and Schwann cells. Mechanistically, integrin β1 (ITGB1) serves as the functional receptor for GC protein in both PDAC and Schwann cells. Targeting the ITGB1‐FAK signaling cascade proves effective in reducing PNI and Schwann cell activation. In KPC (Pdx‐Cre; LSL‐Kras^G12D+^; LSL‐Trp53^R172H/+^) mice and orthotopic xenografts model, GC silencing and ITGB1 blockade both efficiently reduce cancer‐nerve interactions and mitigate PDAC progression. Clinically, GC protein, ITGB1, and phosphorylated‐FAK are positively associated with the severity of PNI in PDAC cases. Collectively, these data demonstrate that GC protein engages integrin receptor signaling to display distinct functions in cancer cells and Schwann cells, thus enabling PNI.

## Graphical abstract

Group‐specific component protein (GC) promotes perineural invasion (PNI) in pancreatic ductal adenocarcinoma (PDAC) by activating Schwann cell dedifferentiation and by enhancing tumor–Schwann cell chemoattraction, independently of vitamin D transport. Integrin β1 (ITGB1) functions as a GC receptor in both cancer cells and Schwann cells. Targeting GC–ITGB1–FAK signaling inhibits PNI and suppresses tumor progression.

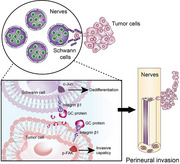

## 1 Introduction

Tumor‐infiltrating nerves represent a crucial component of the tumor microenvironment, significantly contributing to tumor growth, distant metastasis, and resistance to chemotherapy.^[^ ^1^, ^2^, ^3^, ^4^ ^]^ Surgical or pharmacological denervation of sympathetic or sensory nerves has been shown to inhibit tumor progression in many cases.^[^ ^3^, ^5^, ^6^ ^]^ Perineural invasion (PNI), whereby tumor cells directly invade or wrap more than 1/3 of the nerves, is critically implicated in tumor progression.^[^ ^7^ ^]^ PNI has been observed in a variety of solid cancers, with the highest incidence occurring in pancreatic ductal adenocarcinoma (PDAC), reaching 80% to 100%.^[^ ^8^, ^9^, ^10^ ^]^ Clinically, PNI serves as a vital factor influencing local recurrence, distant metastasis, and diminished patient survival rates.^[^ ^11^ ^]^ Additionally, tumor associated pain caused by PNI significantly reduces the quality of life, further worsening the prognosis.^[^ ^12^, ^13^ ^]^ Therefore, elucidating the mechanisms by which cancer cells invade nerves is an essential step toward enhancing treatment strategies for PDAC.

Schwann cells are the main glial cells in the peripheral nervous system, providing support and protection for nerves by wrapping around them to form the myelin sheath, which is vital for neural homeostasis, repair, and regeneration.^[^ ^14^, ^15^, ^16^ ^]^ During nerve repair, Schwann cells undergo demyelination and transform into reparative Schwann cells through a process termed dedifferentiation, in which Schwann cells regain the ability to proliferate and migrate.^[^ ^17^ ^]^ Meanwhile, dedifferentiated Schwann cells re‐express the proteins that were lost during the myelinating differentiation program, such as c‐Jun^[^ ^18^, ^19^ ^]^ and contributes to axon regeneration.^[^ ^20^ ^]^ Schwann cells are involved in cancer progression by adopting a dedifferentiated phenotype, similar to their response during nerve repair.^[^ ^21^ ^]^ The dedifferentiated Schwann cells promote PNI by enhancing cancer cell motility through mechanisms involving pushing, squeezing, or pulling cancer cells, and this process depends on the presence of c‐Jun.^[^ ^22^ ^]^ Importantly, Schwann cells have been shown to emerge around pancreatic intraepithelial neoplasia (PanIN).^[^ ^23^ ^]^ In contrast to the traditional concept of PNI, which emphasizes the invasion of nerves by actively invading cancer cells, this observation suggests that it is the nerves and their inherent cells, rather than the cancer cells, that migrate first to initiate PNI.^[^ ^24^ ^]^ However, the mechanism of how Schwann cells are transformed into a dedifferentiated phenotype in the PDAC remains unclear.

Group specific component protein (GC), also known as vitamin D‐binding protein,^[^ ^25^ ^]^ is not only a vitamin D carrier but also participates in actin scavenging, fatty acid binding, chemotaxis, and macrophage activation.^[^ ^26^, ^27^, ^28^, ^29^, ^30^ ^]^ GC protein is associated with susceptibility or resistance to various chronic diseases, including osteoporosis, diabetes, thyroid autoimmunity, and chronic obstructive pulmonary disease.^[^ ^31^, ^32^, ^33^ ^]^ In addition, GC protein has been implicated in promoting epithelial ovarian cancer progression via regulating the insulin‐like growth factor 1/AKT pathway.^[^ ^34^ ^]^ In a nested case‐control study, a protective link was observed between serum GC protein and PDAC, particularly among men with elevated 25‐hydroxyvitamin D concentrations.^[^ ^35^ ^]^ However, a larger cohort study reported no overall correlation between serum GC levels and PDAC,^[^ ^36^ ^]^ highlighting the ongoing debate regarding the impact of GC protein on PDAC risk and prognosis. Specifically, there has been no research delving into the precise molecular mechanisms through which GC protein influences the progression of PDAC.

In this study, we first analyzed the differentially expressed genes associated with PNI in PDAC and found that GC protein can facilitate PNI independent of its vitamin D transport function, utilizing both an in vitro dorsal root ganglion (DRG) co‐culture model and an in vivo sciatic nerve model. Subsequently, integrin β1 (ITGB1) was identified as the functional receptor that enhances the invasiveness of PDAC cells toward nerves. Furthermore, we demonstrated that GC protein activates Schwann cells by inducing an ITGB1‐dependent dedifferentiation reprogramming, characterized by increased cell proliferation, enhanced cell motility, upregulation of c‐Jun protein, and promoted neurite outgrowth. Finally, we assessed the therapeutic potential of targeting GC‐ITGB1 in the KPC (*Pdx1*‐Cre; LSL‐*Kras* ^G12D/+^; LSL‐*Trp53* ^R172H/+^) mice and orthotopic KPC1199‐derived pancreatic tumor models. Collectively, as a secreted protein, promotes pro‐invasive phenotypes in PDAC cells and induces dedifferentiation in Schwann cells, respectively, thus favoring PNI in PDAC.

## 2 Results

### 2.1 GC Protein is Associated with PNI in PDAC

**Figure 1:**
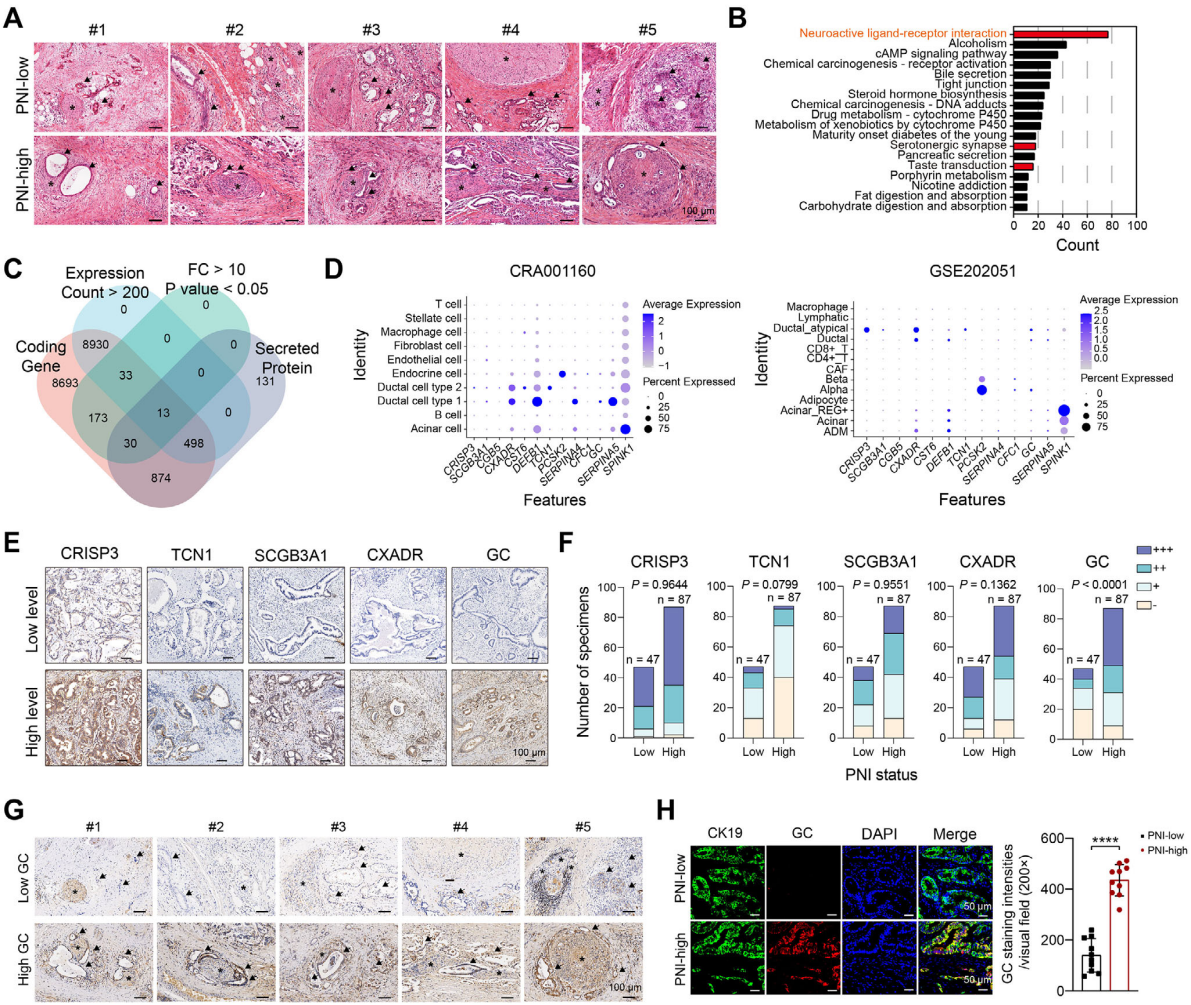
GC protein is a key factor involved in the PNI of PDAC. A) Representative H&E images showed the nerves in PNI‐low and PNI‐high PDAC samples; the asterisk indicates nerves, arrow represent cancer cells, scale bar, 100 µm. B) Kyoto Encyclopedia of Genes and Genomes (KEGG) annotation of differentially expressed genes (DEGs) between PNI‐low and PNI‐high group. C) Venn diagram showed the filter criteria of the candidate DEGs between the PNI‐low and PNI‐high groups. D) Single cell RNA sequencing analysis showed the expression pattern of *CRISP3, SCGB3A1, CGB5, CXADR, CST6, DEFB1, TCN1, PCSK2, SERPINA4, CFC1, GC, SERPINA5*, and *SPINK1* in human PDAC tissues. Data were obtained from CRA001160 and GSE202051. E) Representative immunohistochemical staining of CRISP3, TCN1, SCGB3A1, CXADR, and GC protein expression level in human PDAC tissues in the Ren Ji cohort, scale bar, 100 µm. F) The association between CRISP3, TCN1, SCGB3A1, CXADR, and GC expression level and PNI degree in PDAC tissue in the Ren Ji cohort (n = 134). The yellow, cyan, turquoise, and purple represented ‐, +, ++, and +++ expression level of indicated proteins, respectively. G) Representative immunohistochemical staining of GC protein in human PDAC tissues from the Ren Ji cohort. The asterisk indicates nerves, and arrow represents cancer cells; scale bar, 100 µm. H) Immunofluorescence analysis showed the expression pattern of GC protein in the PNI‐low and PNI‐high human PDAC tissues. Scale bar, 50 µm. ^****^ *P* < 0.0001.

To determine the molecular events related to PNI, we generated a PDAC cohort (Ren Ji cohort, n = 134) and determined the PNI status of each case. Based on the frequency and severity of neural invasion, we evaluated the whole slide H&E images of PDAC cases and divided the samples into two groups: PNI‐low (n = 47) and PNI‐high (n = 87). **Figure** **1**A shows the representative H&E images of PNI‐low and PNI‐high samples. Then, we conducted RNA sequencing analysis on 10 PDAC cases, comprising 5 PNI‐low and 5 PNI‐high samples. The Kyoto Encyclopedia of Genes and Genomes (KEGG) annotation of the differentially expressed genes identified from the comparison between the PNI‐low and PNI‐high groups revealed significant enrichment in neuroactive ligand‐receptor interactions (Figure 1B). Following a thorough analysis and screening utilizing the Venn diagram tool, 13 genes were identified that met the following criteria: these genes are protein‐coding, demonstrate an expression count exceeding 200 in TCGA PAAD tissues, exhibit significant overexpression in the PNI‐high group, and are known to encode secretory proteins (Figure 1C). Single‐cell sequencing analysis of two independent PDAC cohorts (CRA00160 and GSE202051) showed that *CRISP3, TCN1, SCGB3A1, CXADR* and *GC* were expressed at higher level in malignant ductal cells compared with other cell types (Figure 1D). Next, we conducted an immunohistochemical analysis and analyzed the relationship between the 5 candidates and PNI status in a large‐scale PDAC cohort (n = 134) (Figure 1E), and found that only GC is associated with PNI status (Figure 1F). Immunohistochemical staining showed significant GC signals in ductal cancer cells (Figure 1G). Co‐immunofluorescence analysis showed that GC signals co‐localized with CK19, suggesting that GC protein mainly expressed by PDAC cells (Figure 1H). Collectively, these results suggest a GC‐PNI connection in PDAC.

### 2.2 GC Protein Promotes PNI Independent of Vitamin D Transport in PDAC

**Figure 2:**
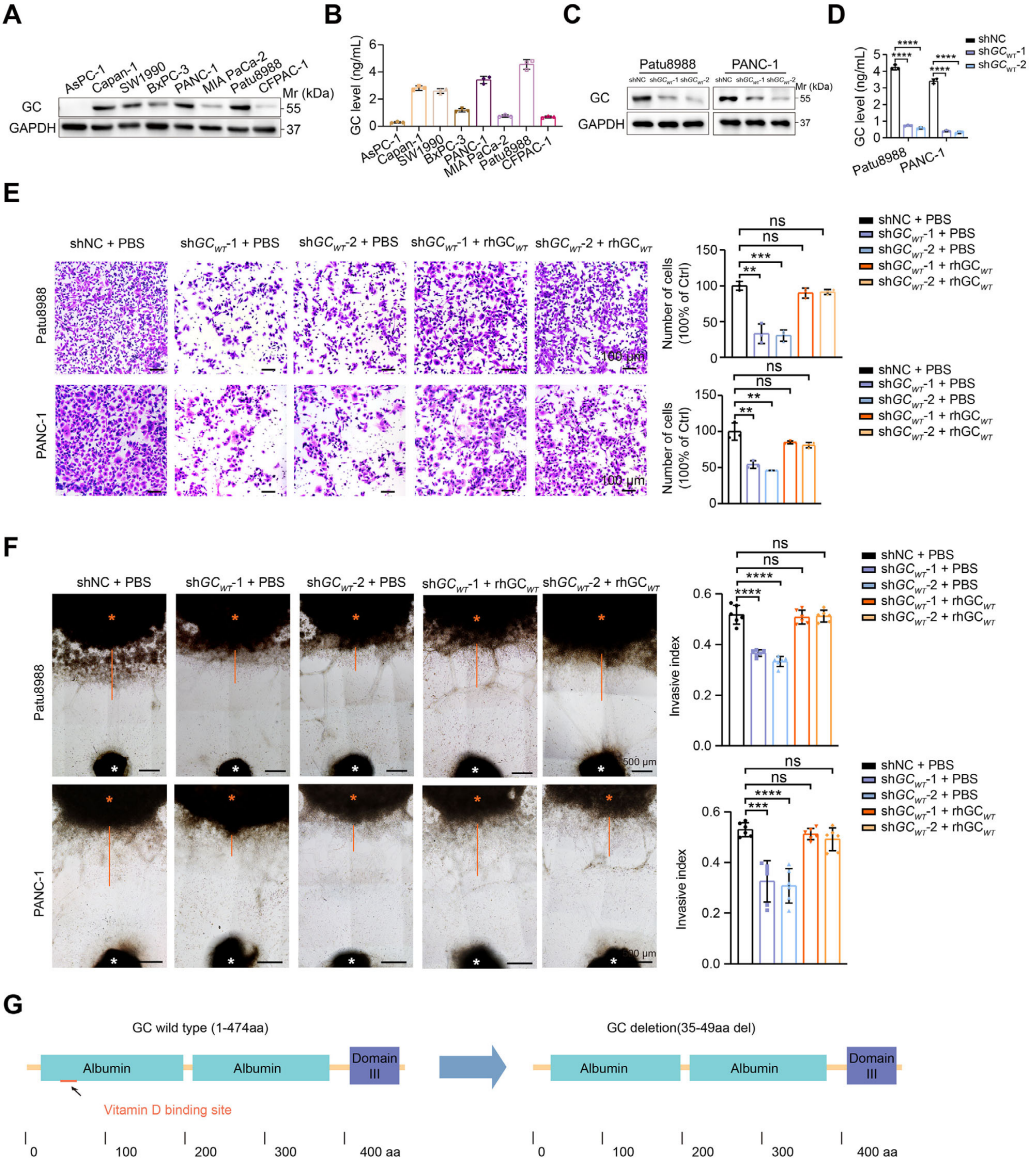
GC protein facilitates cancer invasiveness toward nerves in PDAC. A) Western blotting showing the expression level of GC protein in PDAC cell lines. B) ELISA analysis (n = 3 per group) of secreted GC protein level in PDAC cell lines. C and D) Western blotting and ELISA analysis (n = 6 per group) showing the knockdown efficiency of GC in Patu8988 and PANC‐1 cells; GAPDH was used as a loading control. E) Transwell assay showed the effects of GC silencing on the invasive ability of Patu8988 and PANC‐1 cells with 50 ng mL^−1^ recombinant human wild type GC protein (rhGC_WT_) or vehicle control (n = 3 per group). Scale bar, 100 µm. F) Short hairpin RNAs negative control (shNC) and shGC_WT_ Patu8988 and PANC‐1 cells stimulated with 50 ng mL^−1^ rhGC_WT_ or vehicle control were co‐cultured with mouse DRG and their invasiveness toward DRG was measured at day 10 (n = 6 per group). Scale bar, 500 µm; orange asterisks represent cancer cells and white asterisks indicate DRG, the invasion index was calculated by dividing the distance traveled by invading cancer cells along the DRG neurites by the total distance measured between the tumor colony and the DRG. G) The protein domains for the wild type GC (GC_WT_) protein and the deletion variant GC (GC_DEL_) protein, which lacks amino acids 35 to 49. In all panels, ns: no significance, ^**^ *P* < 0.01, ^***^ *P* < 0.001 ^****^ *P* < 0.0001.

To clarify the biological functions of GC protein, we examined GC expression in 8 PDAC cell lines by western blotting and ELISA experiments (**Figure** **2**A,B). Patu8988 and PANC‐1 cells, which exhibit relatively high GC protein expression, were selected for subsequent loss‐of‐function studies. Two short hairpin RNAs (shRNAs) targeting GC effectively reduced GC protein levels, as evidenced by both western blotting and ELISA (Figure 2C,D). Functionally, knockdown of GC expression had minimal effects on the proliferative potential of PDAC cells (Figure S1A,B, Supporting Information), but markedly reduced their invasive ability, as evidenced by Transwell assay and the DRG coculture assay (Figure 2E,F; Figure S1C, Supporting Information). To test whether GC functions in a secreted manner, we stimulated short hairpin wild type GC (sh*GC_WT_*) PDAC cells with recombinant human wild type GC (rhGC_WT_) proteins. The result showed that rhGC_WT_ protein remarkably promoted sh*GC_WT_* PDAC cell invasive ability (Figure 2E), and sh*GC_WT_* PDAC cells stimulated with rhGC_WT_ protein exhibited greater invasiveness toward DRG neurites compared to control cells (Figure 2F). To assess the role of GC in PNI in vivo, we employed the murine sciatic nerve model of PNI and found that GC knockdown significantly inhibited the invasive ability of Patu8988 cells along the sciatic nerve (Figure S1D,E, Supporting Information). The assessment of sciatic nerve function scores revealed that mice injected with GC knockdown Patu8988 cells in the sciatic nerve experienced only partial paralysis over a three‐week period, indicating a reduced impact of nerve invasion on limb function (Figure S1F, Supporting Information). These results indicate that the introduction of rhGC_WT_ leads to the restoration of invasiveness in sh*GC_WT_* PDAC cells, implying that extracellular GC proteins play a role in PNI.

The classical role of GC protein is responsible for transporting vitamin D.^[^ ^25^ ^]^ To investigate whether GC promotes PNI in a manner dependent on vitamin D, we constructed a human deletion GC recombinant protein (rhGC_DEL_) that lacks the vitamin D binding region, specifically the N‐terminal amino acids 39 to 45 (Figure 2G). The diminished invasive capacity of PDAC cells resulting from GC knockdown was significantly restored upon stimulation with rhGC_DEL_ (Figure S2A, Supporting Information). Additionally, using the DRG coculture model, we demonstrated that rhGC_DEL_ effectively rescued the invasive ability of sh*GC_WT_* PDAC cells toward DRG neurites (Figure S2B, Supporting Information). These results suggest that GC protein enhances PNI independently of its role on vitamin D transport.

### 2.3 GC Protein Facilitates the Dedifferentiation of Schwann Cells Independent of Vitamin D Transport

**Figure 3:**
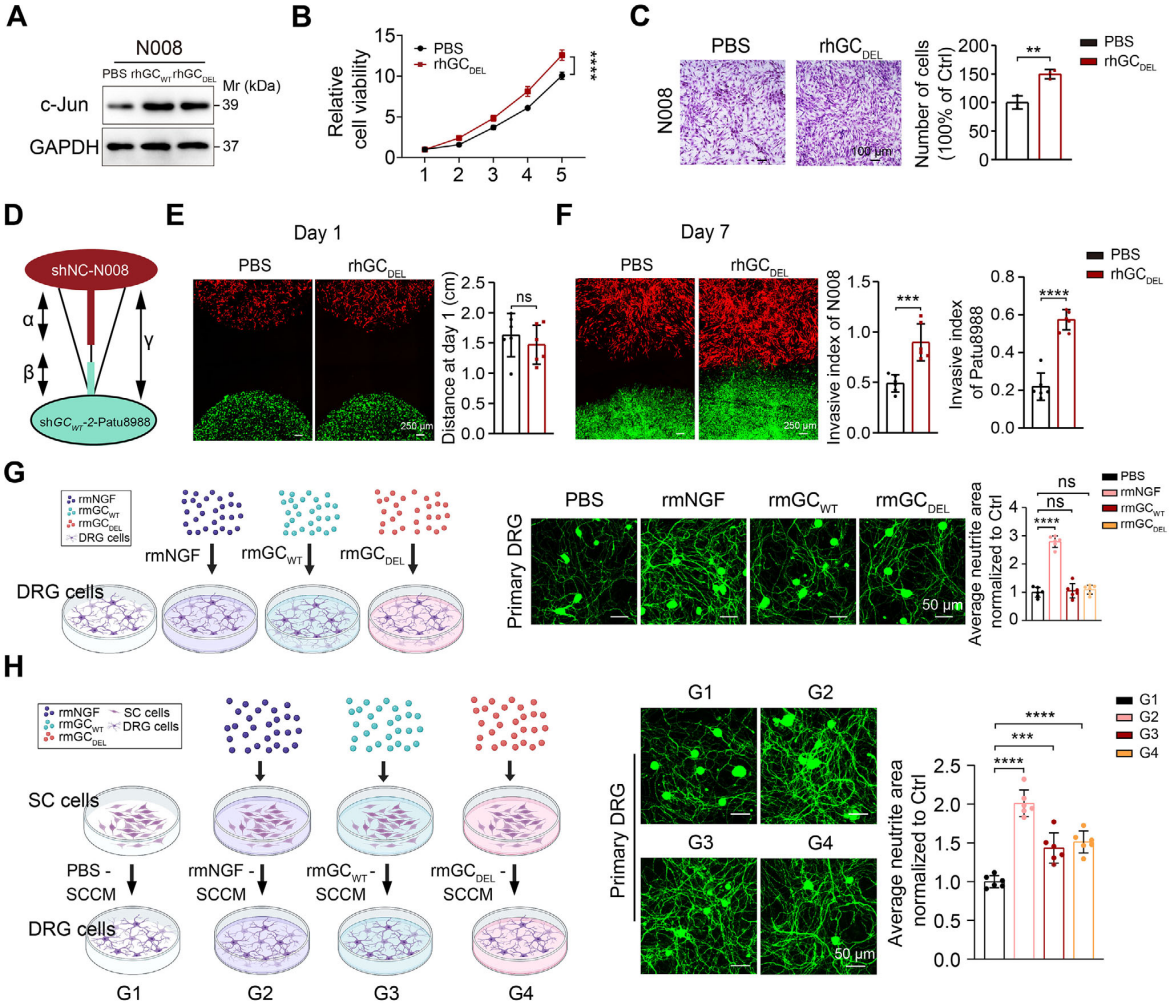
GC protein facilitates Schwann cell dedifferentiation independent of vitamin D transport. A) Western blotting shows the change of c‐Jun following stimulation of PBS, rhGC_WT_ or rhGC_DEL_. B) CCK8 assay showed the proliferation capacity of N008 cells upon treatment of PBS or rhGC_DEL_ (n = 6 per group). Values as mean ± SD and compared by two‐way ANOVA multiple comparisons with Tukey's method. C) Transwell assay showed the migratory capacity of N008 cells with stimulation of PBS or rhGC_DEL_ (n = 3 per group). D) The schematic model depicted the placement of N008 cells transfected with shNC‐RFP lentivirus (shNC‐RFP‐N008) and Patu8988 cells transfected with sh*GC_WT_*‐2‐GFP lentivirus (sh*GC_WT_*‐2‐GFP‐Patu8988) within Matrigel. E) The representative confocal images of PBS and rhGC_DEL_ N008 and Patu8988 in Matrigel invasive model at first day and quantification of the distance between Patu8988 and N008 (n = 6 per group), scale bar, 250 µm. F) Representative images of PBS and rhGC_DEL_ shNC N008 and sh*GC_WT_*‐2 Patu8988 in Matrigel invasive model on the seventh days and quantification of N008 or Patu8988 cell invasion into Matrigel in the presence of PBS or rhGC_DEL_, (n = 6 per group). Invasion index was calculated by dividing the distance of N008 or PDAC cell invading in Matrigel (α/β) by the total distance measured between the PDAC cell and the Schwann cell (γ). G) Effects of recombinant mouse GC (rmGC_WT_ and rmGC_DEL_, 50 ng mL^−1^) proteins on the neurite outgrowth of primary DRG neurons (n = 6 per group). The left panel illustrated the experimental treatment paradigm, the central panel displayed representative βIII‐tubulin immunofluorescence images, and the right panel presented quantitative analysis results. Recombinant mouse NGF protein (rmNGF) protein as a positive control. H) The neurite outgrowth of primary DRG neurons upon stimulation with conditioned medium (CM) from rmGC_WT_ and rmGC_DEL_ (50 ng mL^−1^) proteins‐stimulated mouse Schwann cells (n = 6 per group). Left, the experimental scheme. Middle, representative immunofluorescence images of βIII‐tubulin. Right, quantitative analysis. The CM from rmNGF protein‐stimulated mouse Schwann cells as a positive control. Scale bar, 50 µm. In all panels, ns: no significance, ^**^ *P* < 0.01, ^***^ *P* < 0.001, ^****^P < 0.0001.

Previous studies have proposed that dedifferentiated Schwann cells in the tumor microenvironment promote PNI by enhancing tumor cell invasiveness, while tumor cells may induce Schwann cell dedifferentiation in a paracrine manner.^[^ ^22^, ^24^, ^37^ ^]^ Therefore, we investigated the functional impact of GC protein on Schwann cells. First, we revealed that the expression of c‐Jun was upregulated upon stimulation with rhGC_DEL_ protein in Schwann cells (**Figure** **3**A). Following this, cell proliferation and transwell assays were performed, showing that stimulation with rhGC_DEL_ protein enhanced the growth and migration of Schwann cells (Figure 3B,C). The lentiviral vectors carrying shNC‐RFP or sh*GC_WT_*‐2‐GFP were transfected into N008 and Patu8988 cells, respectively. Subsequent analysis using a Matrigel co‐culture system revealed that rhGC_DEL_ treatment enhanced the invasion ability of PDAC and Schwann cell, and increased the mutual chemoattraction between sh*GC_WT_*‐2 Patu8988 and shNC N008 (Figure 3D–F). Given that dedifferentiated Schwann cells facilitated axonal regeneration following neural injury,^[^ ^20^ ^]^ and acknowledging the positive correlation between neural density and PDAC progression,^[^ ^38^ ^]^ we subsequently assessed the potential effects of GC protein on DRG neurite outgrowth. Immunofluorescence staining of DRG axons with βIII‐tubulin (a pan‐neuronal marker) demonstrated that recombinant mouse GC protein (rmGC_WT_ and rmGC_DEL_, 50 µg mL^−1^) protein exhibited no direct neurotrophic effect on primary mouse DRG neurons (Figure 3G). Strikingly, conditioned medium (CM) from rmGC_WT_ and rmGC_DEL_ protein‐primed Schwann cells significantly potentiated neuritogenesis (Figure 3H). Collectively, these data demonstrated that GC protein potentiate Schwann cell dedifferentiation through a vitamin D‐independent mechanism, manifested by upregulated c‐Jun expression, enhanced proliferative capacity, potentiated migratory activity, and facilitated DRG neurite outgrowth.

### 2.4 ITGB1 Serves as a Functional Receptor for GC Protein

**Figure 4:**
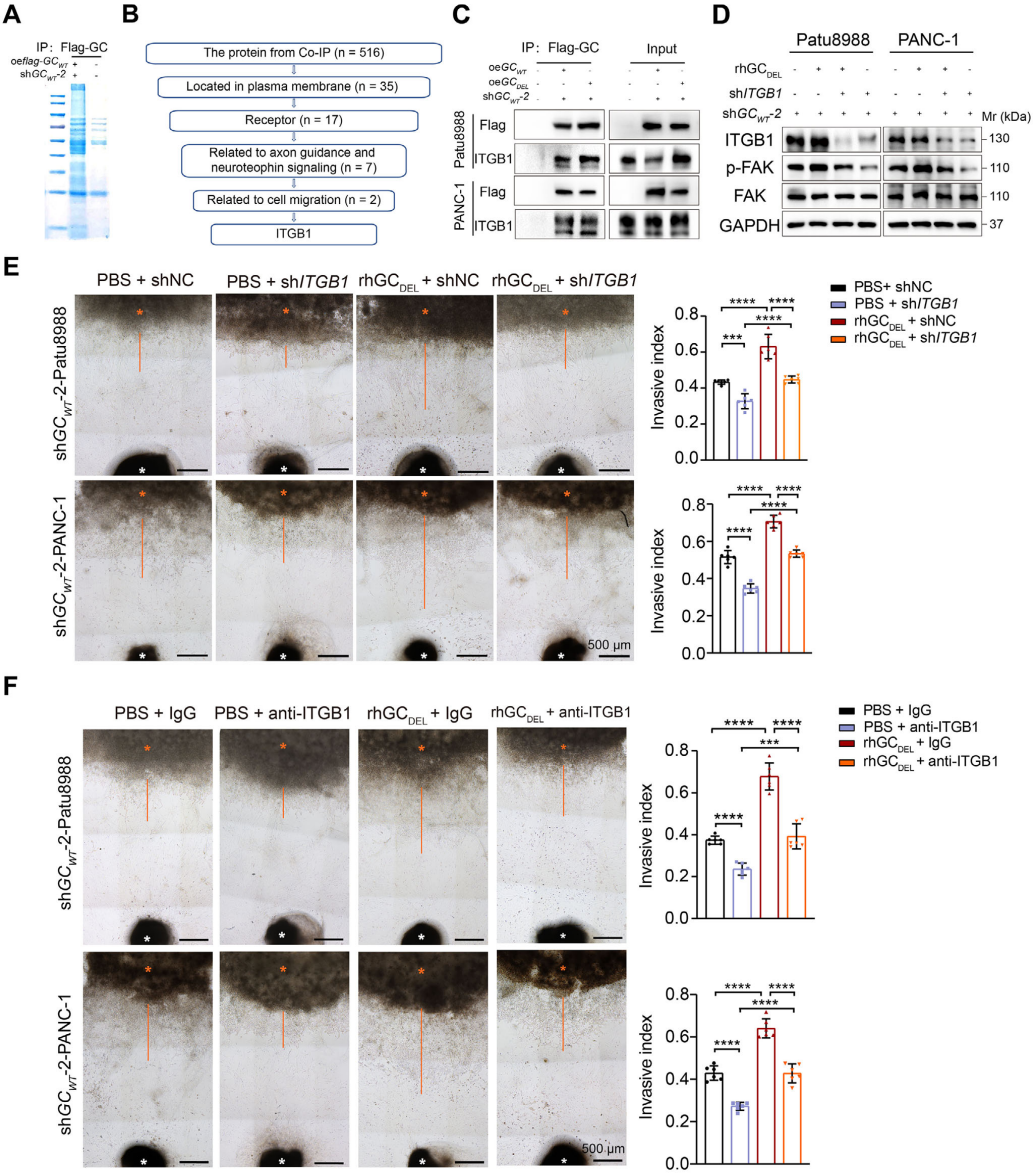
GC protein promotes PDAC cell invasiveness via interaction with ITGB1. A) Coomassie blue staining was performed using Flag antibodies followed by mass spectrometry detection. B) The screening steps for potential interacting receptors of GC protein. C) Flag pulldown of sh*GC_WT_*‐2 Patu8988 and PANC‐1 cells overexpressing Flag‐*GC_WT_* or Flag‐*GC_DEL_*, the wild type cells were employed as a negative control. The precipitates were analyzed by immunoblot with indicated antibodies. D) Western blotting analysis showed the phosphorylation of FAK protein in shNC or sh*GC_WT_*‐2 Patu8988 and PANC‐1 cells upon treatment with rhGC_DEL_. E) DRG coculture assay showing the effects of ITGB1 knockdown on the invasion index of sh*GC_WT_*‐2‐Patu8988 and PANC‐1 cells toward nerves on the presence or absence of rhGC_DEL_ (n = 6 per group). F) DRG coculture assay showed the effect of rhGE_DEL_ on sh*GC_WT_*‐2‐Patu8988 and PANC‐1 cells toward nerves upon treatment with ITGB1 neutralizing antibody (n = 6 per group). In all panels, ^***^ *P* < 0.001, ^****^ *P* < 0.0001.

Considering that GC protein is a secreted protein, we aimed to identify its potential functional receptors. First, the Flag‐GC_WT_ overexpression plasmid was transfected into sh*GC_WT_* Patu8988, and Flag antibody was used for immunoprecipitation (**Figure** **4**A). Following that, the immunoprecipitated proteins were identified using liquid chromatography tandem mass spectrometry. As a result, there are 17 interacting proteins located on the plasma membrane that could potentially function as receptors (Figure 4B). Our data demonstrated that CM derived from rmGC_WT_ and rmGC_DEL_ protein‐primed Schwann cells significantly enhanced the neurite growth of DRG neurons. Neurotrophic factors and axon guidance genes, known for their essential roles in nerve recruitment and axonogenesis, have been shown to play a critical role in modulating PNI in various cancers.^[^ ^39^ ^]^ Therefore, we performed Gene Set Enrichment Analysis (GSEA) using RNA‐seq data from PDAC cases obtained from the TCGA database. This analysis identified seven candidate genes (including ANXA1, CD44, EHD1, ERC1, EZR, ITGB1, and SLC2A1) that exhibit a positive association with axon guidance and neurotrophic signaling pathways (Figure S3A–G, Supporting Information). Given that GC protein facilitates the invasiveness of PDAC cells, we further filtered our data and found that ANXA1 and ITGB1 are related to the epithelial cell migration pathway (Figure S3H, Supporting Information). In addition, analysis on data from Human Protein Atlas dataset demonstrated that ITGB1 expressions were observed on both neuronal cells and glial cells, while ANXA1 was almost not expression on those cells (Figure S3I, Supporting Information). Therefore, ITGB1 was selected for the next step for research.

To determine whether the interaction between GC and ITGB1 is dependent on the vitamin D binding region, Flag‐GC_WT_ and Flag‐GC_DEL_ plasmids were transfected into sh*GC_WT_*‐2 Patu8988 and PANC‐1 cells, which displayed significant knockdown efficiency (Figure 2C). Co‐immunoprecipitation (Co‐IP) experiments revealed a physical interaction between GC and ITGB1 that is independent of the vitamin D binding region (Figure 4C). Although current research primarily associated GC protein functionality with vitamin D transport,^[^ ^25^ ^]^ this study specifically examined its vitamin D independent mechanisms. The results of Figure 2 revealed that rhGC_WT_ and rhGC_DEL_ exhibited equivalent enhancement of tumor cell perineural invasion. The preserved oncogenic activity despite structural disruption of vitamin D‐binding domains confirmed that the functions of GC protein on promoting PNI independently of vitamin D transport. Consequently, we only employed the rhGC_DEL_ protein for subsequent pathway validation. In sh*GC_WT_*‐2 Patu8988 and PANC‐1 cells, western blotting demonstrated that stimulation with rhGC_DEL_ significantly induced phosphorylated FAK, a classical downstream molecule of ITGB1, and this positive regulation was inhibited by the knockdown of ITGB1 (Figure 4D). Moreover, Transwell assay showed that the increased invasive capacity of sh*GC_WT_*‐2 PDAC cells afforded by GC protein was eliminated by ITGB1 knockdown or neutralization of ITGB1 with a blocking antibody (Figure S4A,B, Supporting Information). In the DRG coculture model, ITGB1 knockdown or blocking inhibited the increased invasive ability of PDAC cells toward DRG neurites induced by rhGC_DEL_ (Figure 4E,F). Using the sciatic nerve model generated with sh*GC_WT_*‐2 Patu8988 cells, we tested the link between GC protein and ITGB1 signaling in vivo. Compared with mice in the control group, mice in groups treated with sh*GC_WT_*‐2 Patu8988 cells overexpressing deletion GC (oe*GC_DEL_*) had increased length of nerve invasion and suffered from progressive ipsilateral hind limb paralysis over two weeks. Strikingly, knockdown of ITGB1 significantly effectively mitigated nerve invasion and improved nerve function caused by oe*GC_DEL_* (Figure S4C–F, Supporting Information). Moreover, the increased invasive capacity of sh*GC_WT_*‐2 PDAC cells and invasiveness toward DRG neurites increased by rhGC_DEL_ were also inhibited by defactinib, a specific inhibitor of FAK (FAKi) (Figure S5A,B, Supporting Information). Under in vivo conditions, defactinib also decreased the distance of sh*GC_WT_*‐2 PDAC cell invasion toward sciatic nerve and alleviated paralysis afforded by overexpression of oe*GC_DEL_* (Figure S5C–E, Supporting Information). Collectively, these findings indicate that GC protein promote PNI through the activation of ITGB1 signaling.

### 2.5 GC‐ITGB1 Axis Facilitates the Dedifferentiation of Schwann Cells

**Figure 5:**
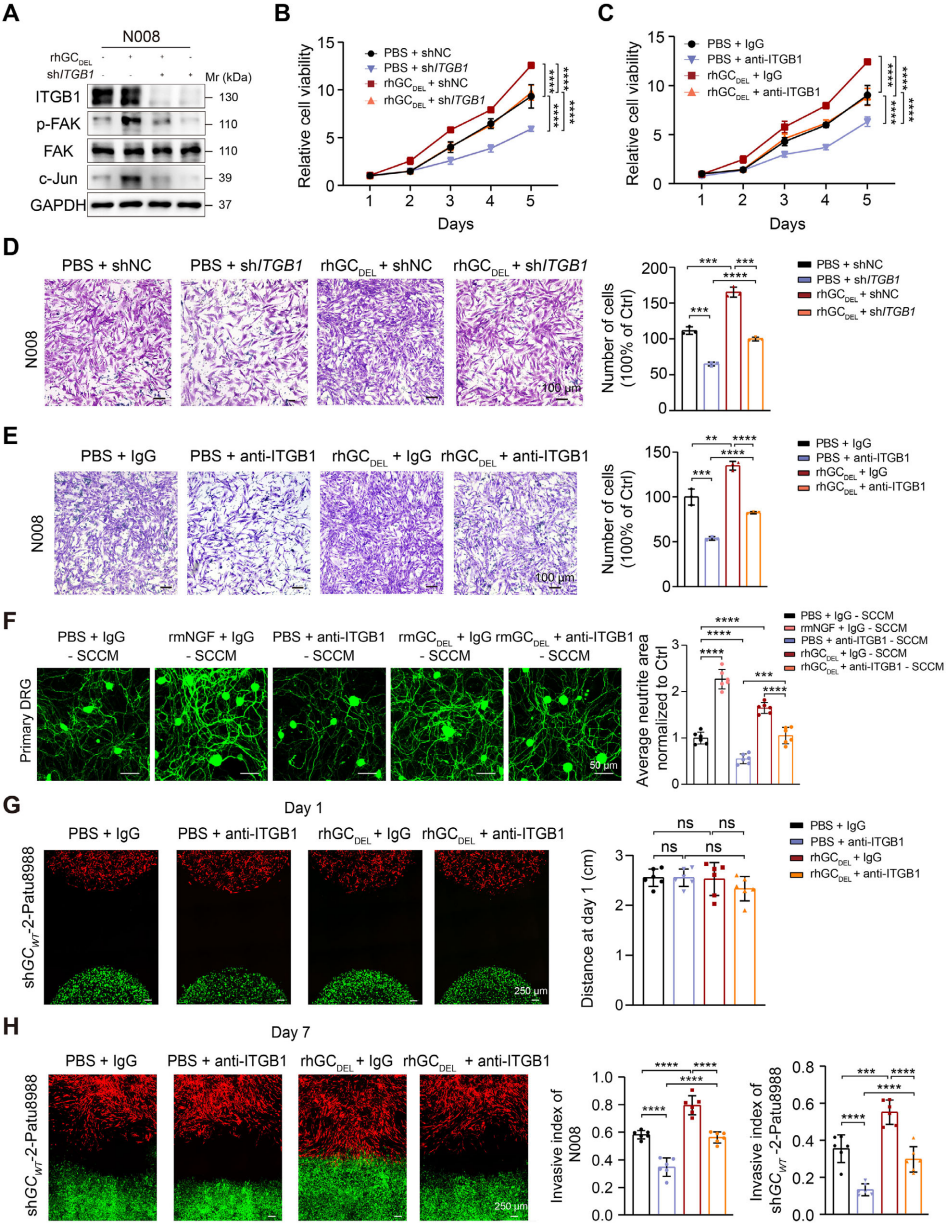
GC protein facilitates Schwann cell dedifferentiation via interacting with ITGB1. A) Western blotting analysis showed the phosphorylation of FAK and c‐Jun expression level in shNC or sh*ITGB1* N008 cells upon stimulated with rhGC_DEL_. B) The proliferation ability of shNC and sh*ITGB1* N008 cell upon stimulated with rhGC_DEL_ or PBS (n = 6 per group). Values as mean ± SD and compared by two‐way ANOVA multiple comparisons with Tukey's method. C) CCK8 assay showed the effect of rhGC_DEL_ on N008 cells upon treatment with anti‐ITGB1 or IgG (n = 6 per group). Values as mean ± SD and compared by two‐way ANOVA multiple comparisons with Tukey's method. D) The migration ability of shNC and sh*ITGB1* N008 cell in the presence or absence of rhGC_DEL_ (n = 3 per group). E) Transwell assay showed the effect of rhGC_DEL_ on the migratory ability of N008 cells upon stimulated with ITGB1 antibody (n = 3 per group). F) Neurite outgrowth of mouse DRG neurons upon treatment with CM from rmGC_DEL_ stimulated‐Schwann cells and anti‐ITGB1 (n = 6 per group). Left, immunofluorescence images of βIII‐tubulin. Right, quantitative analysis. The CM from rmNGF protein‐stimulated mouse Schwann cells as a positive control. Scale bar, 50 µm. G) Representative confocal images of N008 cells transfected with shNC‐RFP lentivirus and Patu8988 cells transfected with sh*GC_WT_*‐2‐GFP lentivirus (in Matrigel invasive model in the presence or absence of rhGC_DEL_ or ITGB1 antibody at first day and quantification of N008 or sh*GC_WT_*‐2 Patu8988 cell invasion into Matrigel upon treatment with rhGC_DEL_ or ITGB1 antibody (n = 6 per group); scale bar, 250 µm. H) Representative images of N008 and sh*GC_WT_*‐2 Patu8988 upon treatment with rhGC_DEL_ or ITGB1 antibody in Matrigel invasive model on the seventh days and quantification of N008 or sh*GC_WT_*‐2 Patu8988 cell migration into Matrigel in indicated groups (n = 6 per group). In all panels, ns: no significance, ^**^ *P* < 0.01, ^***^ *P* < 0.001, ^****^P < 0.0001.

We previously found ITGB1 acted as the functional receptor of GC protein in PDAC cells. Therefore, next, we verified whether the function of rhGC_DEL_ in Schwann cells is dependent on ITGB1. Co‐IP revealed the physical interaction between GC and ITGB1 in N008 cells, whose binding sequences were not included in the vitamin D binding domain (Figure S6A, Supporting Information). Compared to the low expressing samples, the patients with high level of GC or ITGB1 trended toward having higher dedifferentiated gene signature score,^[^ ^40^ ^]^ which is based on analysis of transcriptome data from PDAC cases deposited in the TCGA database (Figure S6B,C, Supporting Information). Previous studies have shown that the classical ITGB1 downstream effectors mainly include FAK, AKT, MAPK, and YAP/TAZ signals.^[^ ^41^, ^42^, ^43^ ^]^ To delineate the predominant pathway through which GC protein orchestrates c‐Jun expression, we implemented a targeted pharmacological inhibition strategy in N008 cells, including FAK inhibitor (defactinib, 5 µm), AKT inhibitor (LY294002, 20 µm), MAPK inhibitor (U0126, 20 µm), and YAP/TAZ inhibitor (verteporfin, 1 µm) in combination with rhGC_DEL_ treatment (50 ng mL^−1^). Western blotting analysis confirmed that FAK pathway was the key signal in upregulation of c‐Jun expression induced by GC_DEL_ protein (Figure S6D, Supporting Information). In addition, ITGB1 silencing decreased the upregulation of p‐FAK and c‐Jun induced by stimulation with rhGC_DEL_ (**Figure** **5**A). The increased expression of dedifferentiated Schwann cells marker (*c‐Jun, GFAP, SOX10, NCAM1, SOX9*, and *L1CAM*) afforded by rhGC_DEL_ protein was also eliminated by ITGB1 knockdown or neutralization of ITGB1 with a blocking antibody (Figure S6E,F, Supporting Information). Moreover, either knockdown of ITGB1 or treatment with ITGB1 blocking antibody attenuated the increased ability of Schwann cell proliferation and migration induced by rhGC_DEL_ (Figure 5B–E). The CM of Schwann cells stimulated by rmGC_DEL_ significantly enhanced neurite growth of DRG neurons, and this paracrine effect was abolished by anti‐ITGB1 neutralization (Figure 5F). In the co‐culture assay, at first day, we observed that the distance between sh*GC_WT_*‐2 Patu8988 and shNC N008 have no statistically significant difference in the indicated group (Figure 5G). On the seventh day, the increased invasive ability of sh*GC_WT_*‐2 Patu8988 and migratory ability of shNC N008, induced by rhGC_DEL_, were largely compromised by the addition of anti‐ITGB1 treatment (Figure 5H). Taken together, the results above suggest that GC protein enhances the dedifferentiation of Schwann cells, composes a reciprocal chemoattraction acting on PDAC and Schwann cells in an ITGB1‐dependent manner.

### 2.6 Inhibition of GC‐ITGB1 Axis Decreases Cancer‐Nerve Interaction and Mitigates PDAC Progression

**Figure 6:**
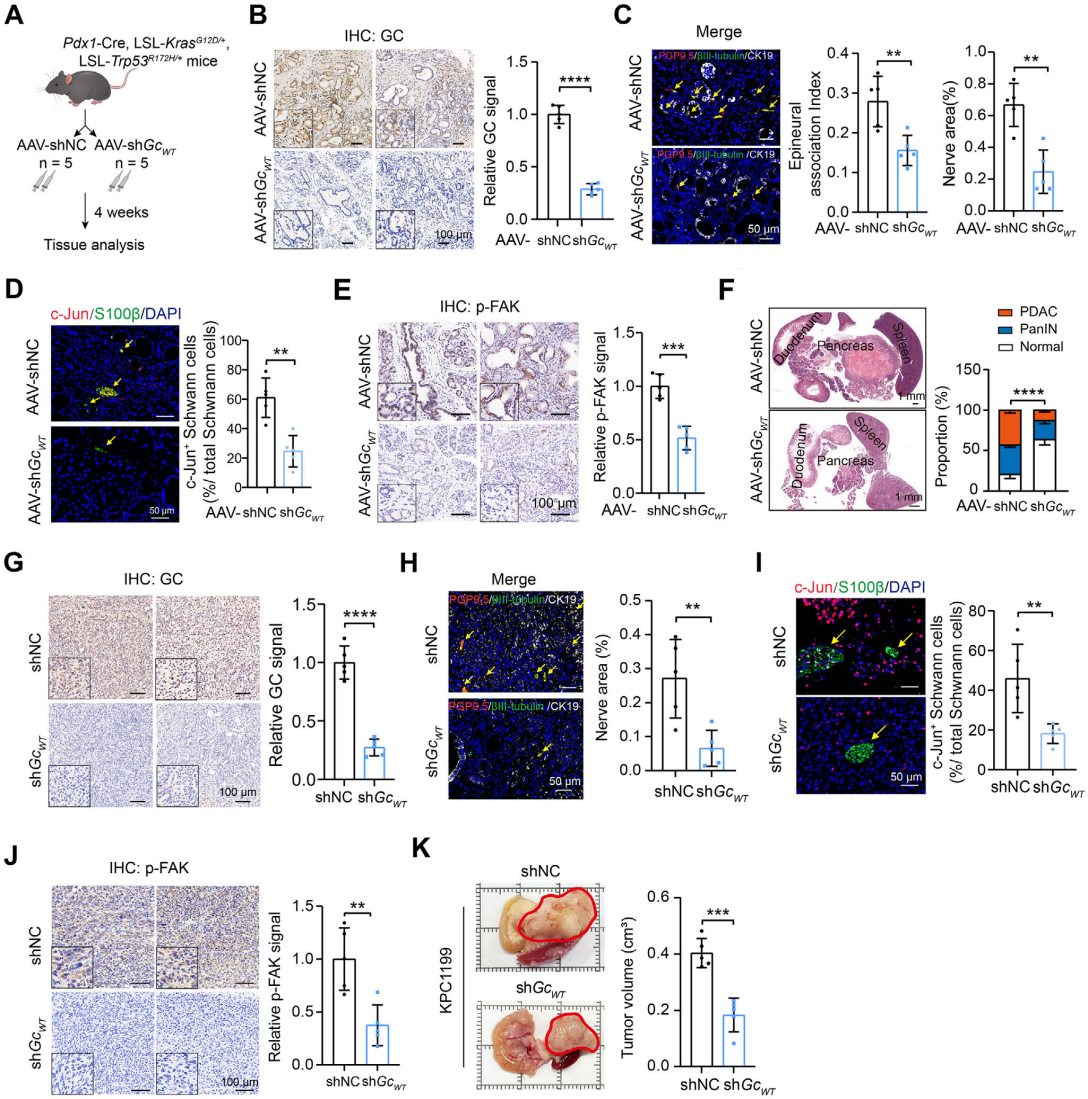
AAV‐mediated GC knockdown inhibits cancer‐nerve interaction and mitigates PDAC progression in vivo. A) Treatment scheme depicting injection of AAV‐shNC and AAV‐sh*Gc_WT_* into the KPC mice (n = 5 per group). B) Immunohistochemical analysis of mouse GC protein in tumor tissues from AAV‐shNC and AAV‐sh*Gc_WT_* KPC mice. Scale bar, 100 µm. C) Comparing the frequency of epineural tumor associations and nerve area in tissue sections from AAV‐shNC and AAV‐sh*Gc_WT_* KPC mice (n = 5 per group). The yellow arrows indicated the nerve. Scale bar, 100 µm. D) Immunofluorescence analysis of c‐Jun expression in Schwann cells within tumor tissues from AAV‐shNC and AAV‐sh*Gc_WT_* KPC mice. The yellow arrows indicated the nerve. Scale bar, 50 µm. E) Immunohistochemical analysis of p‐FAK expression in tumor tissues from AAV‐shNC and AAV‐sh*Gc_WT_* KPC mice. F) Representative H&E images of pancreatic sections from AAV‐shNC and AAV‐sh*Gc_WT_* KPC mice illustrate the differences in tissue morphology between the two groups. The accompanying bar graph quantifies the areas occupied by PDAC and PanIN lesions in both AAV‐shNC and AAV‐sh*Gc_WT_* KPC mice. Scale bar, 1 mm. G) Immunohistochemical validation of GC protein knockdown efficiency in PDAC tissues from shNC and sh*Gc_WT_* orthotopic PDAC models. Scale bar: 100 µm. H) Representative immunofluorescence imaging and quantitative analysis of nerve area in tumor tissues from shNC and sh*Gc_WT_* orthotopic PDAC models. The yellow arrows indicated nerve. Scale bar: 50 µm. I) The expression level of c‐Jun in Schwann cells within tumor tissue following shNC and sh*Gc_WT_* intervention. The yellow arrows indicated nerve. Scale bar: 50 µm. J) Phosphorylation status of FAK in tumor cells from shNC and sh*Gc_WT_* orthotopic PDAC model. Scale bar: 100 µm. K) Representative macroscopic imaging (left) and volumetric quantification (right) of orthotopic tumors in shNC and sh*Gc_WT_* groups. In all panels, ^**^ *P* < 0.01, ^***^P < 0.001, ^****^ *P* < 0.0001.

To gain further insights into the influence of GC proteins on PNI in vivo, we genetically silenced GC genes by infecting KPC mice with adeno‐associated virus (AAV)‐mediated shRNA. KPC mice, which faithfully recapitulate human PDAC, were selected when bearing 5–8 mm tumors, as characterized through palpation. The mice were then randomly divided into two groups: AAV‐shNC and AAV‐sh*Gc_WT_*. Four weeks later, the mice were sacrificed, and the pancreas was harvested for histopathological analysis (**Figure** **6**A). GC protein was significantly downregulated by AAV‐mediated shRNA in the tumor tissues of KPC mice, as evidenced by immunohistochemical analysis (Figure 6B). Although KPC mice do not show classical perineural invasion as observed in human PDAC,^[^ ^7^ ^]^ epineural tumor associations (ETAs) are widely present in KPC tumors. The frequency of ETAs and tumor innervation was significantly mitigated by GC knockdown in KPC tumors (Figure 6C). Immunofluorescence analysis revealed significant downregulation of c‐Jun expression in Schwann cells following GC knockdown (Figure 6D). Histopathologic analysis showed that the expression of p‐FAK in tumor cells was markedly decreased by GC knockdown (Figure 6E). Consistent with the reduction of GC proteins, morphometric quantification of the total surface area occupied by normal tissue, PanIN lesions, and PDAC revealed a decrease in both PDAC and PanIN lesions in the AAV‐sh*Gc_WT_* groups (Figure 6F). Utilizing orthotopic xenografts model established with shNC or sh*Gc_WT_* KPC1199 cells, mice were sacrificed at day 35 (Figure 6G). Given that all infiltrated nerves in this model represent ETAs, quantitative assessment demonstrated markedly suppressed neural density in sh*Gc_WT_* groups (Figure 6H). Concomitant reductions of c‐Jun in Schwann cell and p‐FAK in tumor cell were validated by Immunofluorescence or immunohistochemistry, respectively (Figure 6I,J). Critically, GC knockdown profoundly inhibited pancreatic tumor progression (Figure 6K). To delineate the functional impact of ITGB1 inhibition on neural invasion in vivo, syngeneic KPC mice received intraperitoneal injections of either IgG isotype control or ITGB1‐neutralizing antibody for four weeks prior to histological assessment (Figure S7A, Supporting Information). Quantitative analysis demonstrated that ITGB1 blockade significantly suppressed ETAs frequency and neural density (Figure S7B, Supporting Information). Compared to IgG treatment, the expression of c‐Jun in Schwann cells and p‐FAK in tumor cells were attenuated in KPC mice upon anti‐ITGB1 treatment (Figure S7C,D, Supporting Information). In addition, ITGB1 inhibition reduced tumor burden (Figure S7E, Supporting Information). The suppression of neural invasion and tumor progression of ITGB1 blockade was reproduced in orthotopic PDAC models (Figure S7F–J, Supporting Information). Collectively, these findings demonstrate that GC‐ITGB1 axis is capable of enhancing cancer‐nerve interactions and accelerating PDAC progression.

### 2.7 Clinical Relevance of the GC Signaling‐PNI Connection in PDAC

**Figure 7:**
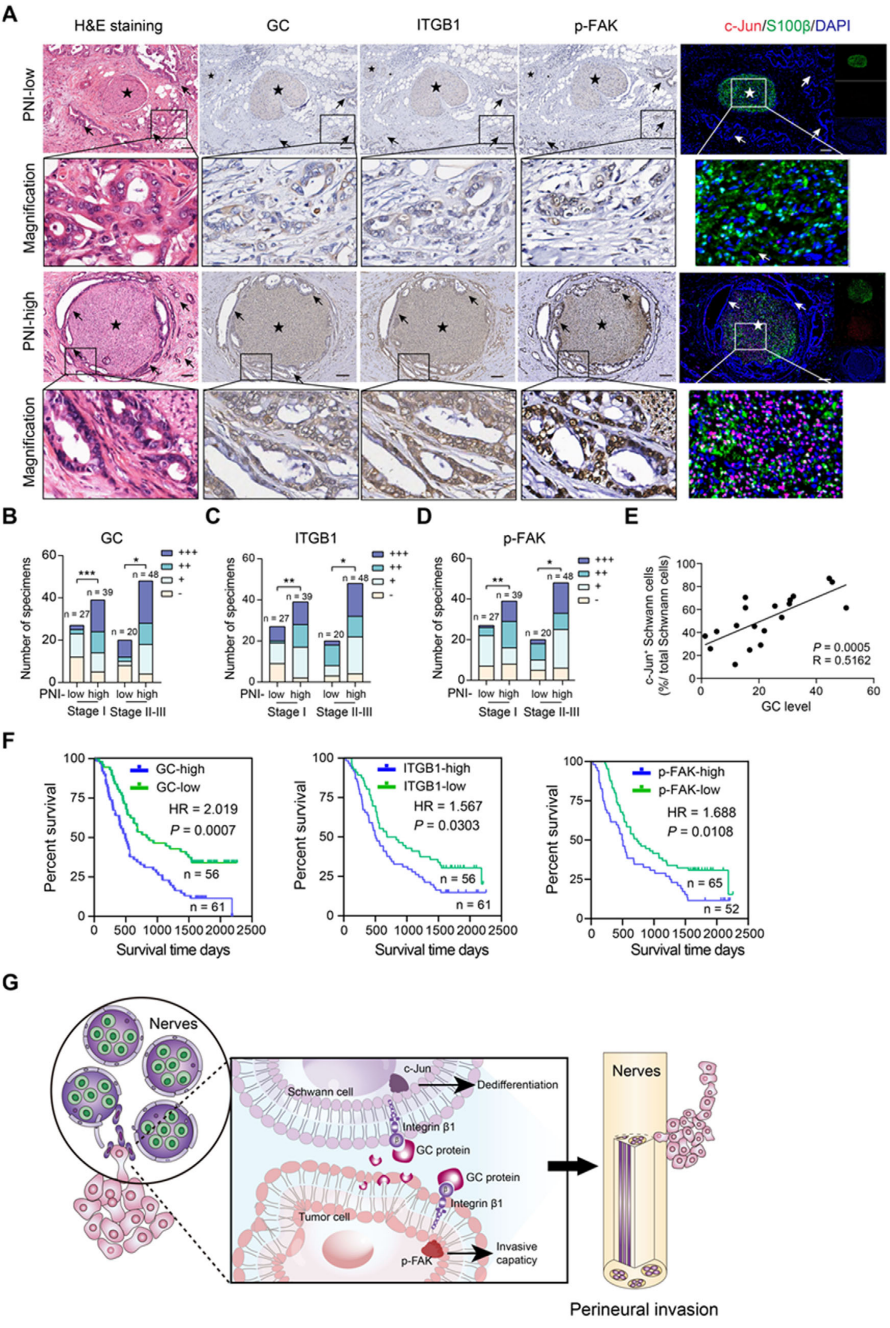
Clinical relevance of the GC signaling‐PNI connection in PDAC. A) Immunohistochemical analysis of GC and ITGB1 in a tissue microarray containing 134 PDAC cases. Immunofluorescence analysis of c‐Jun of S100β‐positive Schwann cells in PDAC sample from Renji cohort. H&E staining showed representative images of PNI. The asterisk indicates nerve, arrow represent cancer cells, scale bar 50 µm. B‐D) Immunohistochemical representation of GC, ITGB1, and p‐FAK expression patterns in TNM stage I and stage II‐III PDAC specimens stratified by high versus low PNI status; “‐”, “+”, “++”, and “+++” represent no staining, weak staining, moderate staining, and strong staining, respectively. ^*^ *P* < 0.05, ^**^ *P* < 0.01, ^***^P < 0.001. E) The association between GC level and c‐Jun expression in S100β‐positive Schwann cells. F) Kaplan–Meier analysis of overall survival related to the expression of GC, ITGB1, and p‐FAK in 117 cases based on Renji cohort. G) The proposed mechanism model of GC protein in facilitating PNI in PDAC. GC protein, primarily derived from PDAC cells, plays a dual effect. On one hand, it directly enhances the invasive potential of tumor cells. On the other hand, it can promote the “dedifferentiation” of Schwann cells and increase the interaction between tumor cells and Schwann cells. This process is mediated by ITGB1 and ultimately leads to the promotion of PNI in PDAC.

To investigate the connection between GC‐ITGB1 signaling and PNI in a clinical setting, we examined a large cohort comprising 134 clinically annotated PDAC cases. The PNI status of each PDAC sample was assessed by two senior pathologists to ensure accuracy. The expression levels of GC, ITGB1, and p‐FAK were analyzed using immunohistochemical method, while c‐Jun expression in nerve tissues was detected through immunofluorescence (**Figure** **7**A). The PDAC patients were stratified by TNM staging into stage I and stage II‐III subgroups. Comparative analysis revealed a significantly higher prevalence of PNI‐high cases in the stage II‐III cohort (70.5%) versus the stage I group (59%). Crucially, GC, ITGB1, and p‐FAK expression levels demonstrated positive correlations with PNI severity in both stage I and stage II‐III subgroups (Figure 7B–D). The intensity of c‐Jun in the Schwann cells within the tumor region was positively related to the GC level (Figure 7E). Kaplan–Meier curve analysis showed that GC, ITGB1, and p‐FAK expression in malignant epithelial cells were associated with worse overall survival in PDAC patients (Figure 7F).

To evaluate the clinical relevance of targeting the GC‐ITGB1 axis, orthotopic xenografts model were established using KPC1199 cells. Mice received treatment of either gemcitabine or DMSO one week after shNC or sh*Gc_WT_* tumor incubation (Figure S8A, Supporting Information). Four weeks later, the mice were sacrificed, and the results demonstrated that GC ablation significantly enhanced tumor sensitivity to gemcitabine, evidenced by reduced tumor volume and Ki67 expression (Figure S8B,C, Supporting Information). Consistently, therapeutic blockade of ITGB1 using neutralizing antibody synergized with gemcitabine, yielding superior tumor suppression (Figure S8D–F, Supporting Information). Collectively, GC‐ITGB1 axis may act as a chemo‐sensitization target in pancreatic adenocarcinoma.

## 3 Discussion

In this study, we propose that GC protein, mainly derived from local cancer cells, promotes PNI in PDAC independently of vitamin D transport. Acting as a ligand for the ITGB1 receptor, GC protein can directly influence cancer cells, enhancing their invasive capabilities toward nerves, promoting dedifferentiation of Schwann cells, and increasing the bidirectional chemoattractant between cancer cells and Schwann cells. The effects of the GC‐ITGB1 axis on both cancer cells and Schwann cells ultimately lead to significant PNI in PDAC (Figure 7G).

The relationship between GC protein and cancer progression have long been studied. Patients with expressing the Gc2 isoform of GC protein benefit from high circulating vitamin D concentration in lung cancer^[^ ^44^ ^]^ or colorectal adenoma.^[^ ^45^ ^]^ Some investigations have observed no association between circulating GC protein level and the risk of urinary bladder cancer^[^ ^46^ ^]^ or prostate cancer.^[^ ^47^ ^]^ However, most of these researches have focused on the predictive value of circulating GC, which acted as vitamin D transporter, with significantly less attention given on the cellular GC protein. Our study further broadens the functions of GC protein in promoting PNI in PDAC. Importantly, GC protein functions as a secretory protein independent on transporting vitamin D.

GC protein has been identified to bind to some receptors located on cell membrane, such as CSPGs,^[^ ^48^ ^]^ low‐density lipoprotein receptor family members,^[^ ^49^ ^]^ CD36,^[^ ^50^ ^]^ CD44, and annexin A2.^[^ ^51^ ^]^ In this study, we further characterized the interaction between GC protein and ITGB1 receptor, which occurs independently of the vitamin D binding site. ITGB1 is an important member of transmembrane glycoprotein receptor integrin family and can regulate the cellular processes through interacting with a various of ligands.^[^ ^42^ ^]^ Indeed, we demonstrated that ITGB1 and the downstream FAK signaling cascades serve as functional mediators in the promotive effects of GC protein on PNI.

During nerve injury, Schwann cells convert into dedifferentiated phenotype. The features of dedifferentiated Schwann cells comprise losing the ability of myelination, becoming more proliferative and motile, forming the Büngner bands to guide axon sprouting, and overexpressing proteins that support axon outgrowth.^[^ ^17^, ^52^, ^53^ ^]^ The Schwann cells response to PNI, which similar to nerve trauma, by converting into dedifferentiated phenotype to promote the progression of cancer.^[^ ^21^, ^54^ ^]^ c‐Jun is an essential transcription factor for Schwann cell dedifferentiation and governs major aspects of Schwann cell response to nerve injury, such as the structure of regeneration tracks, the direct interaction between axons and Schwann cells, and myelin clearance.^[^ ^55^ ^]^ In PNI, the expression of c‐Jun in Schwann cells significantly increases. Moreover, Schwann cells promote cancer cell invasion in a c‐Jun dependent manner.^[^ ^22^ ^]^ The expression of ITGB1 increases in Schwann cells in the sciatic nerve after crush and plays an important role in axon‐Schwann cell interaction and axonal regeneration.^[^ ^56^ ^]^ Consistent with these findings, we revealed that the GC‐ITGB1 axis promotes the dedifferentiation of Schwann cells and facilitates mutual chemoattractant signaling between cancer cells and Schwann cells by upregulating the expression of c‐Jun.

There are several limitations to our study. First, the absence of ideal mouse models for PNI presents a significant challenge in identifying key molecules involved in the development of PNI. The sciatic nerve model utilized in our study fails to accurately replicate the natural nerve innervation of the pancreas, while the KPC model demonstrates epineural tumor associations but does not exhibit a typical PNI phenotype. Second, the regulatory mechanisms by which the GC‐ITGB1 axis influences the expression of c‐Jun in Schwann cells remain inadequately explored, warranting further investigation to fully understand this interaction and its implications in PNI. Third, in this study, while we employed primary human Schwann cells for in vitro functional analysis of their impact on PDAC progression, technical constraints precluded validation using patient‐derived Schwann cells. Moreover, patient‐derived PDAC organoid models were not incorporated into the experimental design. Fourthly, while this study established ITGB1 as the functional receptor mediating GC protein induced PNI in PDAC, the potential receptor roles of other integrin family members, including but not limited to ITGA2, ITGB3, and ITGB5, remained neither investigated nor excluded.

In summary, our study demonstrates that GC protein enhances the invasive potential of PDAC cells and promotes the activation of Schwann cells. These effects are mediated by ITGB1, ultimately facilitating PNI. Targeting the GC‐ITGB1 axis presents a promising strategy to inhibit cancer‐nerve interactions and impede PDAC progression. Our findings offer theoretical insights and highlight potential therapeutic targets for addressing PNI in PDAC.

## 4 Experimental Section

### Cell Culture and Reagents

Human pancreatic cancer cell lines AsPC‐1 (RRID: CVCL_0152), BxPC‐3 (RRID: CVCL_0186), CFPAC‐1 (RRID: CVCL_1119), PANC‐1 (RRID: CVCL_0480), and MIA PaCa‐2 (RRID: CVCL_0428), SW1990 (RRID: CVCL_1723) were obtained from the National Collection of Authenticated Cell Cultures, Chinese Academy of Science (Shanghai, China). Capan‐1 (RRID: CVCL_0237) and Patu8988 (CVCL_1846) were purchased from American Type Culture Collection (ATCC, Manassas, VA, USA). The murine KPC1199 cell line was generously provided by Professor Jing Xue of the Stem Cell Research Center at Ren ji Hospital, Shanghai Cancer Institute, Shanghai Jiao Tong University School of Medicine. The human primary Schwann cell N008 was kindly gifted by Professor Zhichao Wang from the Department of Plastic and Reconstructive Surgery at Shanghai Ninth People's Hospital, Shanghai Jiao Tong University School of Medicine, which was extracted from the human sciatic nerve. Cell line authentication was performed via short tandem repeat (STR) profiling, with all cultures confirming mycoplasma‐free status using the MycoAlert Detection Kit (Lonza) within three months post‐resuscitation. All PDAC cell lines were cultured in a culture medium according to ATCC protocols and supplemented with 10% fetal bovine serum (FBS) and penicillin (100 units mL^−1^) and streptomycin (100 µg mL^−1^). N008 cells were cultured in primary Schwann cell media (iCell bioscience, Shanghai, China). All cells were identified by short tandem repeat (STR) and did not contain mycoplasma. All cells were incubated at 37 °C with 5% CO_2_. Human recombinant protein (rhGC_WT_ and rhGC_DEL_) was purchased from GL biochem (Shanghai, China). Defactinib (a highly potent FAK inhibitor, S7654), LY294002 (a widely utilized PI3K pathway inhibitor, S1105), U0126 (a specific MAPK inhibitor, S1102), and verteporfin (a classical YAP/TAZ signaling inhibitor, S1786) were purchased from Selleck (Shanghai, China).

### Transfection

Patu8988, PANC‐1, and N008 cells were transfected with lentivirus for silencing GC and ITGB1 (GenePharma, Shanghai, China) or overexpressing GC_WT_ and GC_DEL_ (OBiO technology, Shanghai, China). For transducing lentivirus, exponentially growing indicated cells were seeded in 6‐well plate. After cell attachment, 10 µL well^−1^ lentivirus suspension mixed with 1 mL well^−1^ culture medium was added in the presence of 5 µg mL^−1^ polybrene (Gene Pharma, Shanghai, China). After 48 h transfection, the puromycin (5 µg mL^−1^) or Blasticidin S (5 µg mL^−1^) were added into culture medium for selecting stable cell line. The sequences of short hairpin RNAs (shRNAs) used in this study were shown as follow: sh‐*GC*‐1: GCTCACAATATGCTGCTTATG; sh‐*GC*‐2:GCTAAGGGCCCTCTACTAAAG; sh‐*ITGB1*: GCCCTCCAGATGACATAGAAA.

### Western Blotting

The proteins were extracted using the IP lysis buffer (G2038, Servicebio, Wuhan, China) with protease inhibitor (P100S, Beyotime, Shanghai, China) and the supernatant was obtained after centrifugation at 12 000 rpm at 4 °C. After mixed with the sodium dodecyl sulfate (SDS) loading buffer, the proteins were separated by 8–12% SDS‐polyacrylamide gel electrophoresis. Then, proteins were transfected onto the nitrocellulose filter membrane (FFN02, Beyotime, Shanghai, China). Afterward, the membranes were blocking with 5% non‐fat milk solution (P0216, Beyotime, Shanghai, China) in Tris‐buffered saline (TBS). Subsequently, the membranes incubated with specific primary antibodies overnight at 4 °C. Then, membranes were washed with TBST three times and incubated with the (horseradish peroxidase) HRP‐linked secondary antibody for 1 h at room temperature. Following incubation with secondary antibody, membranes were washed with TBST three times and detected by the chemiluminescence system (BIO‐RAD). The primary antibodies were listed as follows: GC (1:1000, Proteintech, 16922‐1‐AP, RRID: AB_10597098), ITGB1 (1:1000, Proteintech, 12594‐1‐AP, RRID: AB_2130085), FAK (1:1000, Cell Signaling Technology, 71433, RRID: AB_2799801), p‐FAK (Tyr397) (1:1000, Cell Signaling Technology, 8556, RRID: AB_10891442), GAPDH (1:10000, Proteintech, 10494‐1‐AP, RRID: AB_2263076), c‐Jun (1:1000, Proteintech, 24909‐1‐AP, RRID: AB_2860574), Flag‐tag (1:1000, Cell Signaling Technology, 14793, RRID: AB_2572291) and HPR‐linked anti‐rabbit IgG (1:5000, Cell Signaling Technology, 7074, RRID: AB_2099233).

### Quantitative Real‐Time PCR

Total RNA was extracted from frozen tissues or cells using TRIzol reagent (Thermo Fisher Scientific, #15596026) and performed reverse transcription according to the instructions of PrimeScript RT reagent kit (RR037B, Takara Bio, Japan). Real time qPCR analysis was utilized with SYBR Green Premix qPCR master mix (B21203, Bimake, China) on a ViiA7 real‐time PCR system (Applied Biosystems, USA). Relative mRNA expression was calculated based on the 2^−ΔΔCt^ method, and the mRNA expression level of 18S was used as a control for analysis. The detailed sequences of the primers used in this study were shown in Table S1 (Supporting Information).

### Co‐IP

The proteins were extracted using the IP lysis buffer (G2038, Servicebio, Wuhan, China) with protease inhibitor (P100S, Beyotime, Shanghai, China) and the supernatant was obtained after centrifugation at 12 000 rpm at 4 °C. The anti‐Flag Magnetic beads (B26101, Selleck, Shanghai, China) were washed with phosphate buffered saline with tween 20 (PBST) three times and then incubated with the cell lysate overnight at 4 °C. The next day, the supernatant was removed. The beads were washed with PBST for three times and resuspended in 60 µL 1× SDS‐PAGE loading buffer (P0015, Beyotime, Shanghai, China) and subjected to Western blot analysis.

### In Vitro DRG Co‐Culture Model

The 4 weeks C57BL/6J mice were euthanatized using carbon dioxide asphyxia and DRGs were isolated from mice as previously described.^[^ ^57^ ^]^ The DRGs were harvested in DMEM containing with 10% FBS and then the signal DRG were implanted on a 24‐well plate in 2.5‐µL drop of growth factor‐depleted Matrigel matrix (#356231, Corning, USA). A total of 3 × 10^4^ PDAC cells were suspended in a 5‐µL Matrigel gel and then plated at ≈1 mm adjacent to the DRG suspension. After the Matrigel polymerization, an additional cell‐free ECM gel was generated to bridge the DRG and cancer cells. Cultures were grown in DMEM containing 10% FBS at 37 °C and 5% CO_2_. On day 5, the medium was changed into DMEM containing 3% FBS to support cell survival. In indicated groups, recombinant human GC protein (50 ng mL^−1^) or anti‐ITGB1 (5 µg mL^−1^, R&D systems, Cat. MAB17781), or defactinib (5 µm, Selleck, Cat. S7654) was added into the medium. The neural invasion index was calculated by dividing the distance traversed by the invading cancer cells along the DRG neurites at day 10 (α) by the total distance measured between the tumor colony and the DRG (β).

### Transwell Invasion Assay

The transwell chambers (8.0 µm, Millipore, USA) were pre‐coated with matrigel (BD Biosciences, Catalog #354234, USA) and then were used in 24‐well plates. The upper chambers were loaded with cancer cells (1 × 10^4^) suspended in 0.2 mL serum‐free medium. For N008 cell, 1 × 10^4^ N008 cells suspended in 0.2 mL serum‐free medium were placed on the upper chambers without Matrigel. The medium containing 10% (v/v) FBS was placed in the lower bottom of each well. In indicated groups, recombinant human GC protein (50 ng mL^−1^) or anti‐ITGB1 (5 µg mL^−1^, R&D systems, Cat. MAB17781), or defactinib (5 µm, Selleck, Cat. S7654) was added into the upper chamber. The non‐invaded cells were scraped off of the superior surface of the membranes at 48 h later. Cells on the lower surface were fixed with 4% paraformaldehyde and then stained with 0.2% crystal violet. The invaded cells were quantified by counting stained cells in 6 random fields under a light microscope.

### Immunohistochemistry (IHC)

IHC was performed as described previously.^[^ ^58^ ^]^ Briefly, paraffin‐embedded tissues sections (5 µm) were deparaffinized with xylene, followed by rehydrated with graded ethanol. Then the sections were placed in boiling citrate buffer for antigen retrieval. Sections were pretreated with 3% hydrogen peroxide solution for blocking endogenous peroxidase. Next, diluted primary antibodies were added to each slice and incubated overnight at 4 °C. The secondary antibody labeled with the HRP were incubated 1 h at room temperature. After undergoing color development with diaminobenzidine, the slides were counterstained by hematoxylin. Finally, all the samples were mounted and photographed by a microscope. The primary antibodies were listed as follows: GC (1:100, Proteintech, 16922‐1‐AP, RRID: AB_10597098), ITGB1 (1:100, Proteintech, 12594‐1‐AP, RRID: AB_2130085), FAK (1:1000, Cell Signaling Technology, 71433, RRID: AB_2799801), p‐FAK (Tyr397) (1:50, Cell Signaling Technology, 8556, RRID: AB_10891442), PGP9.5 (1:4000, Proteintech, 66230‐1‐Ig, RRID: AB_2881621), CRISP3 (1:500, Proteintech, 14847‐1‐AP, RRID: AB_1607510), TCN1 (1:100, Proteintech, 16078‐1‐AP, RRID: AB_2878215), SCGB3A1 (1:100, Novus, NBP1‐36983, RRID: AB_2183538), CXADR (1:100, Proteintech, 11777‐1‐AP, RRID: AB_2087443), and HPR‐link secondary antibodies (anti‐Rb, 1:5000, ab205718, RRID: AB_2819160, anti‐Ms, 1:5000, ab205719, RRID: AB_2755049, Abcam, USA). The intensity was quantified with Image J sofeware. Staining intensity was graded on a scale of 0 (absent), 1 (week), 2 (moderate), and 3 (strong). The composite H‐score (range 0–300) was generated by multiplying the proportion of cells exhibiting each intensity level by its corresponding score and summing these products. Scores were categorized as “‐” (negative, 0–50), “+” (low positive, 51–100), “++” (moderate positive, 101–200), or “+++” (strong positive, 201–300). Expression levels were subsequently stratified into low (≤100) and high (>100) categories. All assessments were independently performed by two board‐certified pathologists under blinded conditions.

### Immunofluorescence

The slides of human PDAC or KPC tumors were treated the same as the immunohistochemical protocol until water‐bath heated in citrate solution (G1202, pH 6.0, Servicebio, Wuhan, China). After antigen retrieval, the slides were blocked with 10% BSA for 1 h at room temperature. Then, slides were incubated with the diluted primary antibodies overnight at 4 °C. The primary antibodies were anti‐GC (1:50, Proteintech, 16922‐1‐AP, RRID: AB_10597098), anti‐CK19 (1:200, Proteintech, 10712‐1‐AP, RRID: AB_2133325), anti‐βIII‐tubulin (1:100, Abcam, ab18207, RRID: AB_444319), anti‐PGP9.5 (1:300, Proteintech, 66230‐1‐Ig, RRID: AB_2881621), anti‐phospho‐c‐Jun (Ser73, 1:500, Cell signaling technology, 3270, RRID: AB_2129575). Next, the species‐specific secondary antibodies (anti‐Rb, 1:5000, ab205718, RRID: AB_2819160, anti‐Ms, 1:5000, ab205719, RRID: AB_2755049, Abcam, USA) were added to each slide and incubated 1 h at room temperature. Finally, slides were covered with mounting medium containing DAPI and images were captured with confocal microscopes (Leica, Germany). Quantitative immunofluorescence analysis was performed by randomly selecting five microscopic fields per section for statistical evaluation. Analysis of fluorescence intensity was conducted using ImageJ software (version 1.54b), with the calculated mean value designated as the representative fluorescence intensity.

### Cell Proliferation Assay

N008 cells (2 × 10^3^ well^−1^) in 100 µL of primary Schwann cell medium were seeded in 96‐well plate. For the indicated group, cells were treatment with rhGC_DEL_ (50 ng mL^−1^) or anti‐ITGB1(5 µg mL^−1^, R&D systems, Cat. MAB17781). Cell counting kit‐8 (10 µL well^−1^, CCK8, Dojindo Molecular Technologies, Kyushu, Japan) reagent mixed with 90 µL well^−1^ serum‐free DMEM was added to each well at 0, 1, 2, 3, 4 days. After incubation for 1 h, the cell absorbance was measured at 450 nm by a microplate reader (Bio‐TEK).

### Enzyme‐Linked Immunosorbent Assay (ELISA)

A total of 1 × 10^6^ PDAC cells /well were seeded in 35 mm dishes overnight. The day after attachment, cells were washed with PBS, and 1 mL serum‐free DMEM was added to each well. After 24 h, the conditional medium was collected and cleared by centrifugation at 1500 × g for 10 min. Then, the concentrations of GC in cell supernatant were evaluated using ELISA kits (R&D System, #DVDBP0B) according to the manufacturer's instructions.

### RNA Sequencing

RNA sequencing was performed by Sinotech Genomics (Shanghai, China). The Fragments Per Kilobase per Million (FPKM) method was used to detect the gene expression. Differential expression analysis for mRNA was performed using R package edgeR. The RNA‐seq data in this study were deposited in the Sequence Read Archive (SRA) repository under accession numbers PRJNA1133919.

### In Vivo Sciatic Nerve Invasion Model

The murine sciatic nerve invasion model was generated to study PNI. BALB/c nude mice (male; 6 weeks old; n = 5 per group) were anesthetized with 2% isoflurane, and their sciatic nerves were surgically exposed by making a 1 cm incision ≈2 mm below and parallel to the femur. Then, 3 µL of 5 × 10^4^ Patu8988 cells from the pellet into a 10 µL syringe were slowly injected into the sciatic nerves under magnification. The size of the engrafted tumors was measured with digital calipers and grown until 100 mm^3^. Mice were randomly assigned to control or experimental groups. pushed into the tumor. To mitigate disparities between groups, mice in the control group were injected with PBS. Defactinib was administered intraperitoneally at 10 mg kg^−1^ three times a week until the end of observation. Nerve function was measured after the injection and weekly thereafter. The sciatic nerve function was scored according to hind limb paw response to the manual extension of the body, from 4 (normal) to 1 (total paw paralysis). Nerve invasion was determined by measuring the distance from the proximal edge of the tumor up to the most distal edge of thickened nerve. At the end of the experiment, mice were sacrificed, and sciatic nerves and tumor tissues were dissected and fixed in 4% paraformaldehyde. When making paraffin sections, the dissected nerves were embedded longitudinally, and the letters P and D were used to indicate the proximal (P) and distal (D) ends of the nerve. H&E staining was performed to determine PNI. Length of invasion was measured up from the distal edge of the tumor to the most distal point where cancer cells were present. For quantification of nerve invasion, three slides with 50 µm intervals were evaluated and a median value was selected. Mice were kept on a 12–12 h light‐dark cycle at 23 ± 1 °C and 40–75% humidity. Mice were fed ad libitum with standard chow and water throughout the experiment. All manipulations were in accordance with the principle of the Helsinki Declaration (No. KY2016‐75) and assigned by the Research Ethics Committee of Shanghai Jiao Tong University (Approval number, 202201427).

### Human PDAC Specimen Collection and Ethics Committee

All human tissues for immunohistochemical analysis were obtained from the Department of Biliary‐Pancreatic Surgery, Ren Ji Hospital, School of Medicine, Shanghai Jiao Tong University. All patients included in the study had complete follow‐up data, such as population characteristics (age and sex), surgical records (tumor size, tumor invasion, and tumor location), pathology reports (tumor stage, lymph node status, and metastasis), and survival times. Written informed consent was obtained from all patients before enrollment. The study was approved by the Ethics Committee of Renji Hospital, School of Medicine, Shanghai Jiao Tong University School of Medicine (approval number RA‐2021‐095).

### Evaluation of PNI

By adopting the Ceyhan score,^[^ ^59^ ^]^ PNI status is divided into low degree and high degree using the frequency of neural invasion and the severity of neural invasion. In detail, the frequency of neural invasion was scored as absent/0, low/1, frequent/2, and excessive/3. The severity of neural invasion was defined as absent/0, perineural/1, and intraneural/2. To calculate the degree of neural invasion, the severity and frequency of neural invasion were multiplied. For each PDAC case, the score of neural invasion severity was calculated by adding the number (n) of nerves with the above‐mentioned severities and dividing it by tissue area, as shown in the following formula: Individual neural invasion severity score = $\frac{n(\mathrm{noninvaded})\;\times \;0+\;n(\mathrm{perineural})\;\times \;1+\;n(\mathrm{intraneural})\;\times \;2}{\mathrm{Tissue}\;\mathrm{area}\;(\mathrm{cm}2)}$. According to the final score, the patients were divided into 2 groups: PNI‐low (score < 1), none to mild PNI; and PNI‐high (score ≥ 1), moderate to severe PNI. Two independent senior pathologists who were unaware of the pathological parameters evaluated the PNI status of each sample.

### AAV‐Mediated GC Knockdown In Vivo

The adeno‐associated virus (AAV), which encoding sh*Gc_WT_* or shNC were purchased from Genechem Co.LTD (Suzhou, China) to silence the mouse GC gene in vivo. The sequence of sh*Gc_WT_* was shown as 5′‐ ATGGACCAGTATACATTTGAA‐3′. The transgenic mouse model KPC (LSL‐Kras^G12D/+^, LSL‐Trp53^R172H/+^, Pdx1‐Cre mice) used in the study were described previously.^[^ ^60^ ^]^ When the pancreatic tumor was ≈5–8 mm by digital palpation, KPC mice were randomly divided into two groups (shNC and sh*Gc_WT_*). KPC mice were given orthotopically injection of AAV‐sh*Gc_WT_* and AAV‐shNC (5 × 10^11^ viral particles mouse^−1^) two times. KPC mice were sacrificed 4 weeks following AAV injection to collect pancreatic tissues for further histopathologic analysis.

### The Orthotopic Xenografts Tumor Models

C57BL/6J adult male mice, aged 6 to 8 weeks were used in this study. A total cell number of 5 × 10^4^ shNC and sh*Gc_WT_* KPC1199 cells in 25 µL DMEM were injected into the head of the pancreas. Mice were randomized into four groups treated with DMSO vehicle or gemcitabine (50 mg kg^−1^). after 7 days post‐surgery. For in vivo ITGB1 blockade, C57BL/6J male mice (6–8 weeks old) received orthotopic implantation of 5 × 10⁴ KPC1199 cells in 25 µL DMEM into the pancreatic head. Seven days post‐surgery, tumor‐bearing mice received intraperitoneal administration of anti‐ITGB1 antibody (10 mg kg^−1^; R&D Systems MAB2405) or IgG control twice weekly. Following four weeks of intervention, tumors were excised and volumetrically assessed using the formula: V = ½ × (length × width^2^).

### Neurite Outgrowth Analysis

Primary DRG neurons were plated on glass‐bottom dishes and maintained in neurobasal medium supplemented with B‐27, 2 mm L‐glutamine, 10% FBS, and 1% antibiotic‐antimycotic. Neuronal cultures were stimulated with Recombinant murine GC proteins (rmGC_WT_ and rmGC_DEL_) or indicated conditioned media. After 48 h, DRG neuron were fixed with 4% paraformaldehyde at RT for 30 min and underwent immunofluorescence staining with anti‐βIII‐tubulin antibody. Confocal microscopy (Leica, TSC SP8) captured randomized fields for quantitative analysis. Neurite outgrowth parameters were determined by normalizing total neurite area (ImageJ v1.54b) to cell counts per field.

### Quantifications of Epineural Tumors Associations (ETAs) in KPC Model

To quantify of ETAs in KPC mice, the whole pancreatic tissues were cut into 5 µm thick sections and stained for the pan neuronal marker PGP9.5. Three sections with 100 µm intervals from each mouse were chosen randomly for quantification of ETAs. The nerve that directly contacts with tumor cells was defined as nerves with epineural tumor associations (ETAs). The index of ETAs was calculated as the number of nerves with ETAs dividing by the number of total nerves. PNI was defined as the invasion of tumor cells into the fiber sheath or the encapsulation of tumor cells around the epineurium more than 33% of its circumference. ETAs refer to tumor cell‐nerve direct interaction. In KPC mice, PNI cannot be found, and only ETAs were observed.

### PanIN Lesions and PDAC Quantification in KPC Mice

Based on the classification consensus, early‐stage pancreatic intraepithelial neoplasia (PanIN): PanIN1A and 1B, late‐stage PanIN: PanIN2/3, and PDAC could be found in KPC mice.^[^ ^61^, ^62^ ^]^ CK19 staining was used to further confirm the presence of PanIN and PDAC. The pancreas adjacent to PanIN lesions or PDAC was considered as normal tissue. For each mouse, 3 sections were sampled 100 µm apart, and 5 random views were taken for counting the number of lesions. The total surface analyzed area of sections was measured for each field of view. The percentage of total analyzed surface area occupied by normal tissue, PanIN lesions, or PDAC were calculated.

### Single Cell RNA‐Seq Data Processing

The processed scRNA‐seq dataset CRA001160^[^ ^63^ ^]^ (including 24 primary PDAC samples and 11 normal samples) and GSE202051^[^ ^64^ ^]^ (including 43 primary PDAC samples) were downloaded from the Genome Sequence Archive and Gene Expression Omnibus, respectively. The Seurat R package was used to analyze the expression of *CRISP3, SCGB3A1, CGB5, CXADR, CST6, DEFB1, TCN1, PCSK2, SERPINA4, CFC1, GC, SERPINA5*, and *SPINK1* in immune cells, pancreatic acinar cells, and stromal cells.

### Statistical Analysis

The statistical analysis was performed using GraphPad Prism 8.0 (La Jolla, CA, USA) and SPSS software 17.0 (IBM. Chicago, IL, USA). Results were analyzed with two‐tailed Fisher's exact test, two‐tailed unpaired Student's *t*‐test, and one‐way ANOVA. Cumulative survival time was calculated by the Kaplan–Meier method and analyzed by the log‐rank test. All quantified data were expressed as mean ± SD. *P* value less than 0.05 was considered statistically significance.

### Ethics

All human PDAC samples were obtained from the Department of Biliary‐Pancreatic Surgery, Ren Ji Hospital, School of Medicine, Shanghai Jiao Tong University. This study was approved by the Research Ethics Committee of Ren Ji Hospital, School of Medicine, Shanghai Jiao Tong University (No. RA‐2021‐095). The animal experiments according to the principle of the Helsinki Declaration (No. KY2016‐75) and assigned by the Research Ethics Committee of Shanghai Jiao Tong University (Approval number, 202201427).

## Conflict of Interest

The authors declare no conflict of interest.

## Author Contributions

S.Z., L.J., S.C., Z.W., Y.Z. contributed equally to this work. S.H.J., J.F.Z., X.M.Y., D.X.L., S.Z., L.J.J., S.Q.C., Z.Q.W., and Y.H.Z. carried out conception and design. S.Z., L.J.J., S.Q.C., Z.Q.W., and Y.H.Z. performed the development of methodology. S.Z., L.J.J., S.H.J., S.Q.C., Z.W.C., Q.L., L.P.H., and H.F.Y. were responsible for the acquisition of data. S.Z., J.F.Z., X.M.Y., L.J.J., Z.Q.W., Y.H.Z., R.H., and S.H.J. carried out the analysis and interpretation of data. S.Z., L.J.J., S.Q.C., Z.Q.W., Y.H.Z., S.H.J., and Y.Z. contributed to writing, reviewing, and/or revision of the manuscript. S.H.J., X.M.Y., J.F.Z., S.Z., L.J.J., S.Q.C., Z.Q.W., and Y.H.Z. provided administrative, technical, or material support. All authors read and approved the final version of the manuscript.

## Supporting information



Supporting Information

## Data Availability

The data that support the findings of this study are available from [Gene Expression Omnibus]. Restrictions apply to the availability of these data, which were used under license for this study. Data are available from the authors with the permission of [Gene Expression Omnibus].
