# Supplementary material for: Vitamin D Binding Protein, a Ligand of Integrin beta 1, Motivates Both Tumor Cells and Schwann Cells to Promote Perineural Invasion in Pancreatic Ductal Adenocarcinoma
Source: Adv Sci (Weinh). 2025 Sep 9;12(44):e11726. doi: 10.1002/advs.202511726 (PMC12667536; doi:10.1002/advs.202511726)
Supplement: Supplementary file 1 — Supporting Information [file ADVS-12-e11726-s001.docx]

**
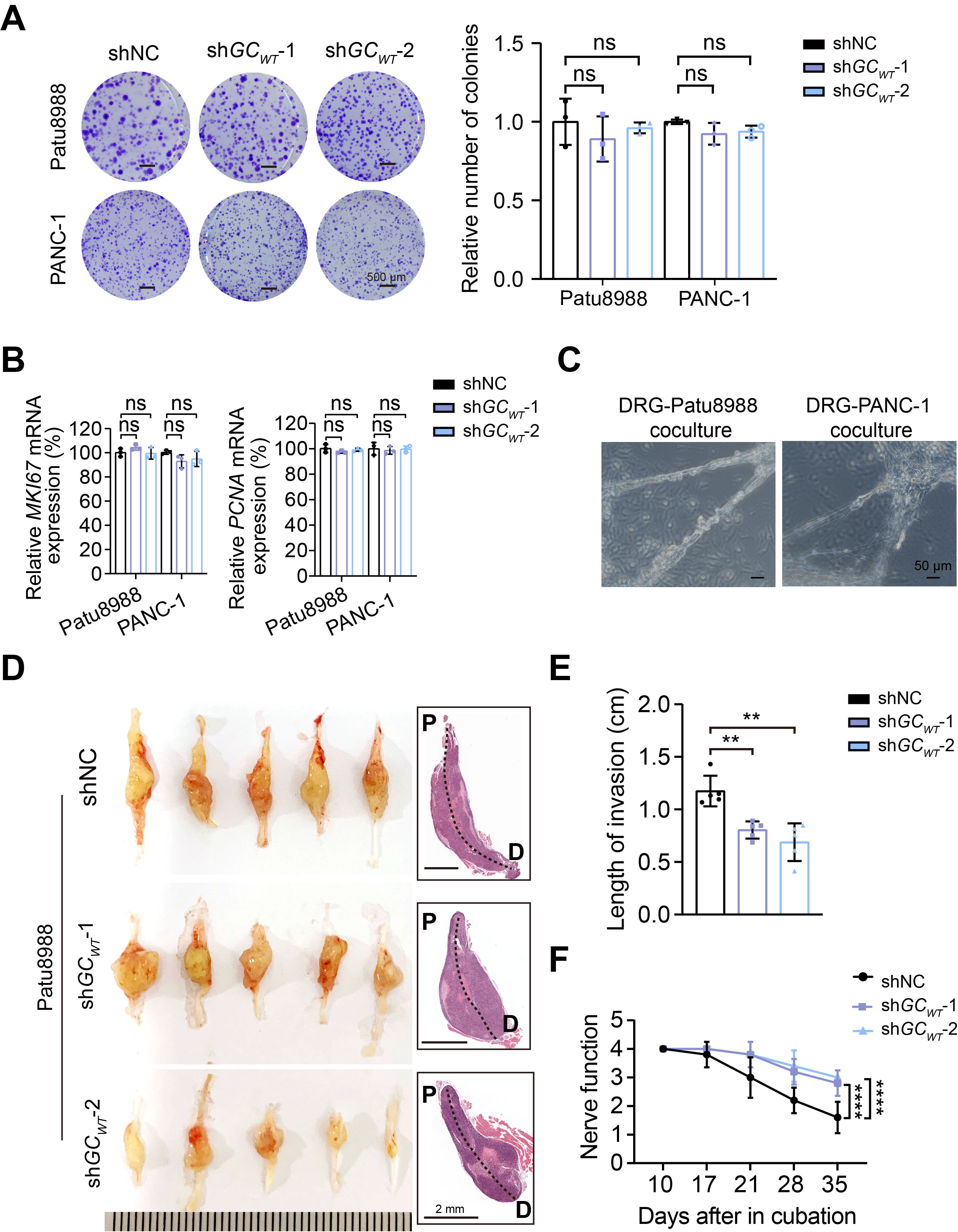
Supplementary information**

**Figure S1.** Effects of GC protein on the proliferation and invasion ability of PDAC cells. A) Plate colony formation assay showed the effects of GC knockdown on the proliferation of Patu8988 and PANC-1 cells (n = 3 per group). Scale bar, 500 μm. B) The effects of GC knockdown on the mRNA expression of *MKI67* and *PCNA* in Patu8988 and PANC-1 cells (n = 3 per group). C) The representative images of cancer cells invasion along the axon that growing from DRG in the DRG coculture assay. Scale bar, 50 μm. D) The image of sciatic nerve invasion was shown in the left panel. Length of nerve invasion within shNC and sh*GC* groups as marked by dotted lines; the letters P and D were used to indicate the proximal and distal ends of the nerve (right panel); scale bar, 2 mm. E and F) The length of sciatic nerve invasion and sciatic nerve function of mice in shNC and sh*GC_WT_* groups (n = 5 per group). In all panels, ns, no significance; ***P* < 0.01, *****P* < 0.0001.


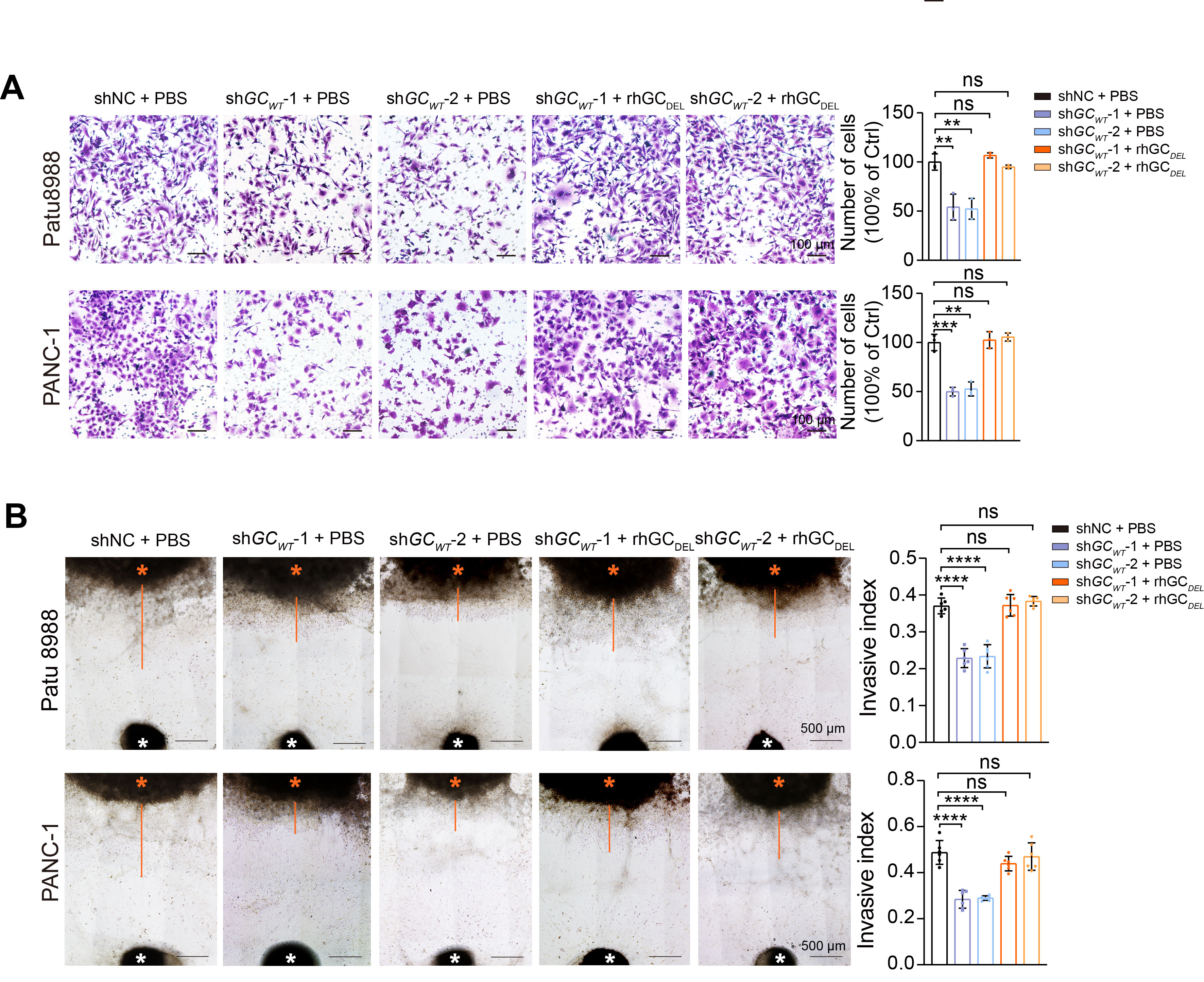


**Figure S2.** GC protein promotes PNI independent of vitamin D transport. A) Transwell assay showed the effects of rhGC_DEL_ (50 ng/mL) on invasive ability in GC knockdown Patu8988 and PANC-1 cells (n = 3 per group). Scale bar, 100 μm. B) DRG coculture assay showing the invasion index of shNC and sh*GC_WT_* Patu8988 and PANC-1 cells towards nerves upon treatment with rhGC_DEL_ (50 ng/mL) or vehicle control (n = 6 per group). Scale bar, 500 μm. In all panels, ns: no significance, ***P* < 0.01, ****P* < 0.001, *****P* < 0.0001.


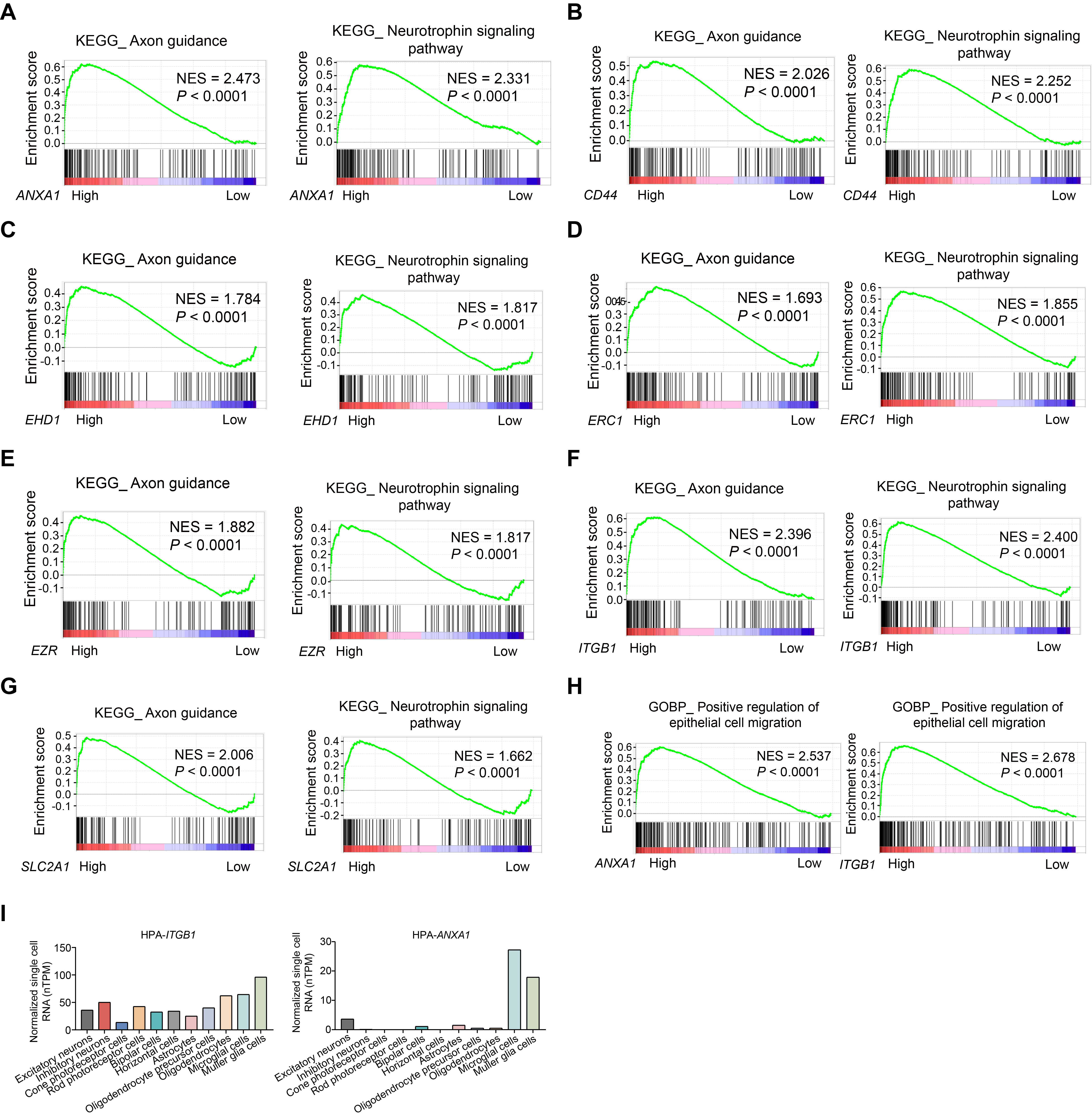


**Figure S3.** The strategy for screening and identifying potential interacting receptors of GC. A-G) Gene set enrichment analysis (GSEA) based KEGG enrichment plots of axon guidance and neurotrophin signaling pathway, grouped by the expression of candidate genes (*ANXA1*, *CD44*, *EHD1*, *ERC1*, *EZR*, *ITGB1*, and *SLC2A1*) group. H) GSEA plot of positive regulation of epithelial cell migration between high or low *AXNXA1* and *ITGB1* groups. The transcriptome data was performed from PAAD cohort in the TCGA database. I) The normalized single cell RNA of ITGB1 and ANXA1 in neuronal cells and glial cells in Human Protein Atlas dataset.

**
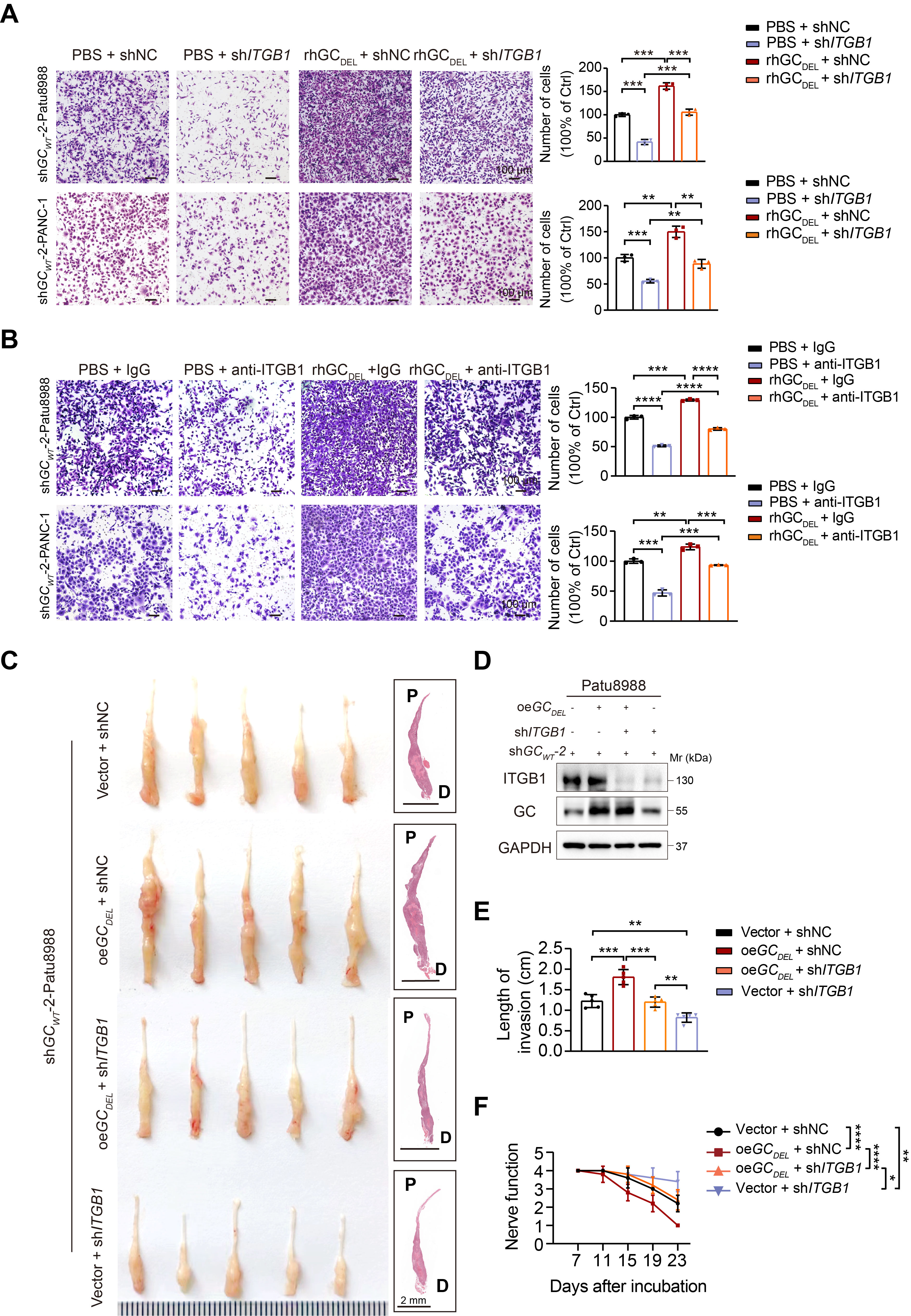
**

**Figure S4.** GC protein promotes PNI via interacting with ITGB1. A) Transwell assay showed the invasion ability of shNC and sh*ITGB1* Patu8988 and PANC-1 cells upon stimulation with rhGC_DEL_ (n = 3 per group). Scale bar, 100 μm. B) Transwell assay showed the effect of rhGC_DEL_ on Patu8988 and PANC-1 in presence or absence of ITGB1 antibody (n = 3 per group). Scale bar, 100 μm. C) The image of sciatic nerve invasion was shown in the left panel. Patu8988 cells (vector + shNC, oe*GC_DEL_* + shNC, oe*GC_DEL_* + sh*ITGB1*, vector + sh*ITGB1*) were injected into the perineurium of sciatic nerve. Length of nerve invasion within indicated groups as marked by dotted lines; the letters P and D were used to indicate the proximal and distal ends of the nerve (right panel); scale bar, 2 mm. D) Western blotting analysis of the efficiency of oeGC_DEL_ overexpression +/- ITGB1 knockdown in Patu8988 cells. E and F) The length of sciatic nerve invasion and sciatic nerve function of mice in indicated groups (n = 5 per group). In all panels, **P* < 0.05, ***P* < 0.01, ****P* < 0.001, *****P* < 0.0001.


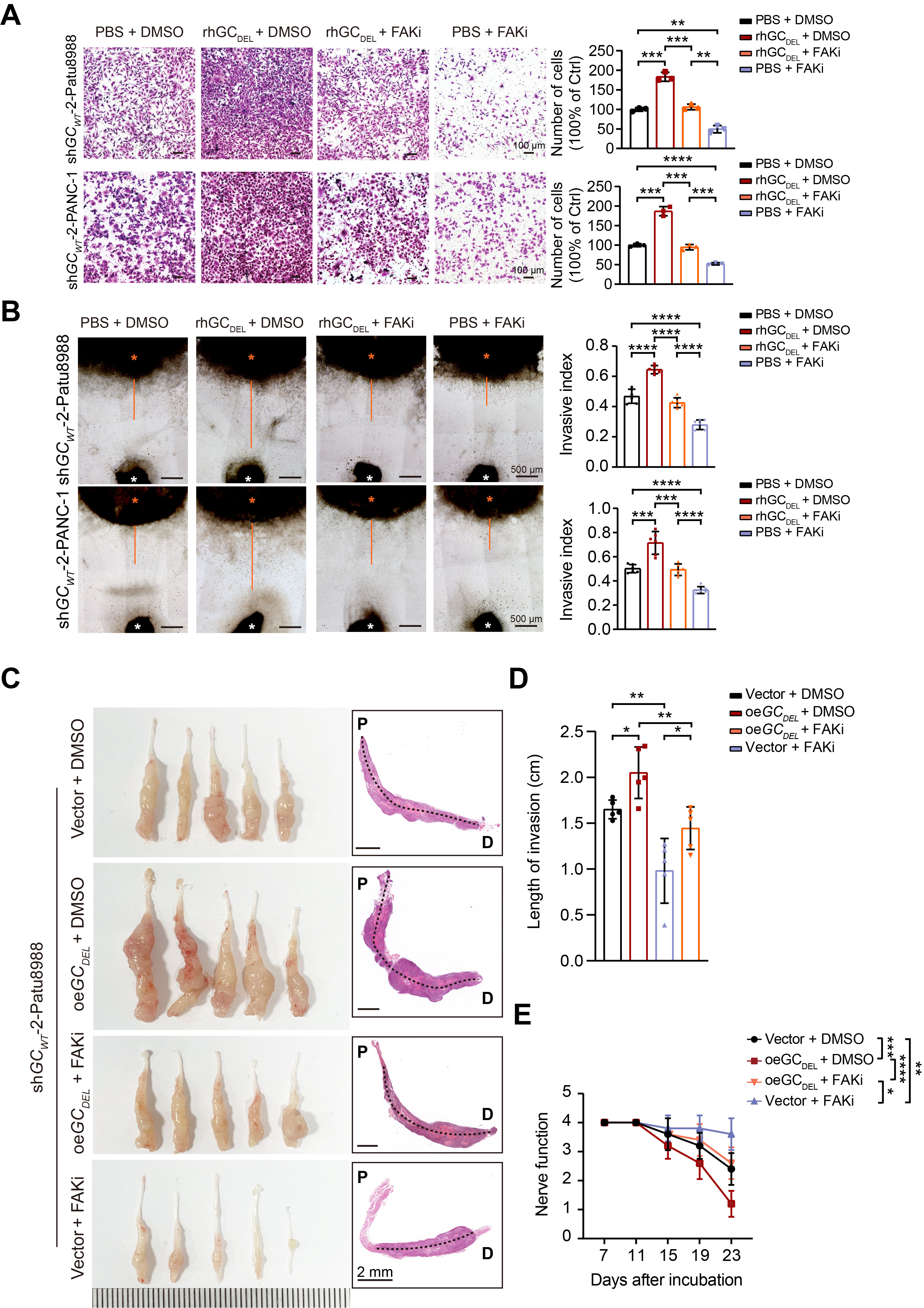


**Figure S5.** FAK inhibitor blocks the promotive effect of GC protein on PNI. A) Transwell assay showed the effect of rhGC_DEL_ on PDAC cells upon treatment with defactinib, a FAK inhibitor (n = 3 per group). Scale bar, 100 μm. B) DRG coculture assay showed the invasion index of Patu8988 and PANC-1 cells towards nerves stimulated with rhGC_DEL_ in presence of absence of defactinib (n = 6 per group). Scale bar, 500 μm. C) The image of sciatic nerve invasion was shown in the left panel. Patu8988 cells (vector and oe*GC_DEL_*) were injected into the perineurium of sciatic nerve following treatment with DMSO or defactinib. Length of nerve invasion within indicated groups as marked by dotted lines; the letters P and D were used to indicate the proximal and distal ends of the nerve (right panel); scale bar, 2 mm. D and E) The length of sciatic nerve invasion and sciatic nerve function of mice in indicated groups (n = 5 per group). In all panels, **P* < 0.05, ***P* < 0.01, ****P* < 0.001, *****P* < 0.0001.


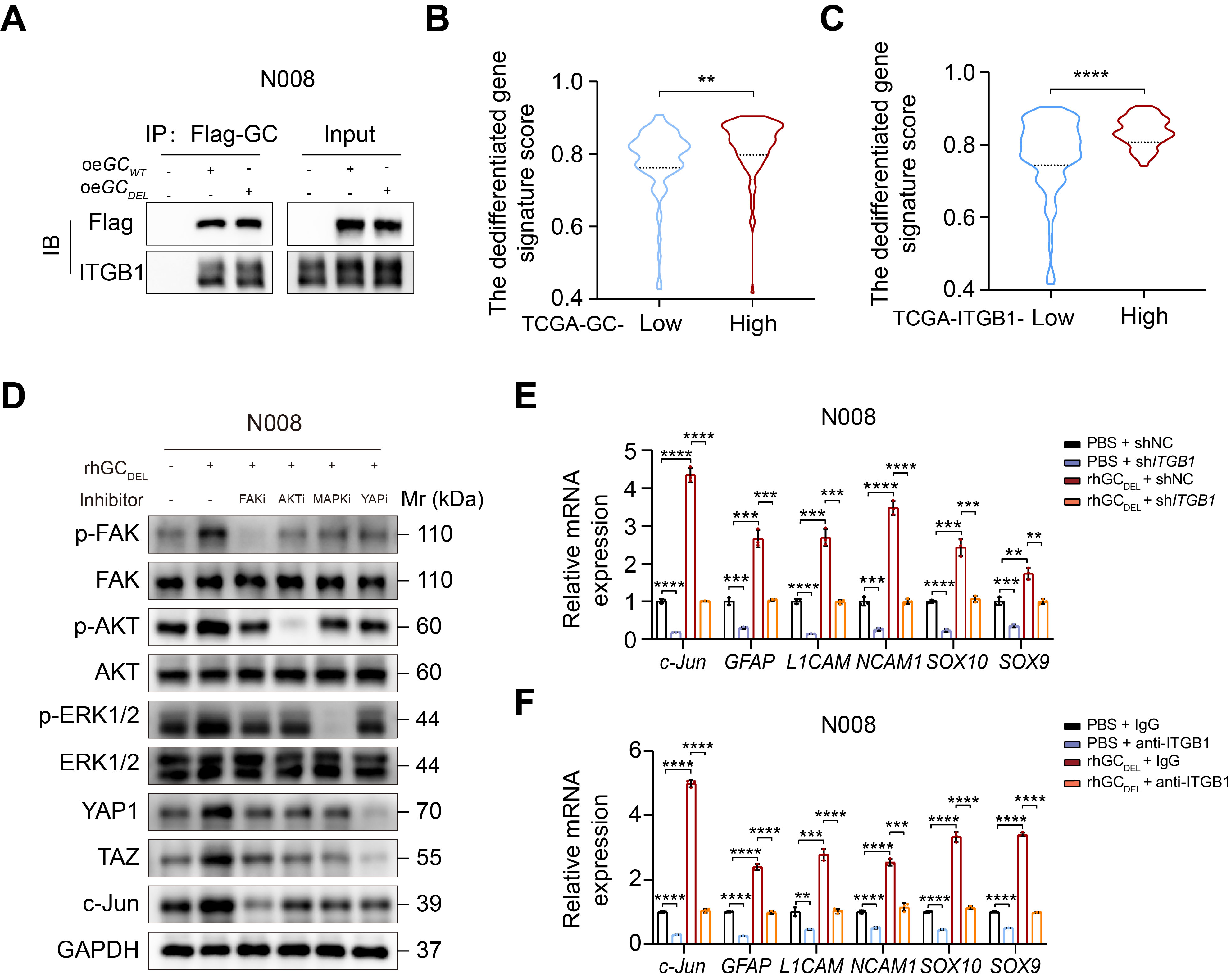


**Figure S6.** Expression of GC and ITGB1 is related to the gene signature of dedifferentiated Schwann cells**.** A) Flag pulldown of N008 cells overexpressing Flag-GC_WT_ or Flag-GC_DEL_, the wild type cells were employed as a negative control. The precipitates were analyzed by immunoblot with indicated antibodies. B and C) The violin diagram shown the difference of dedifferentiated gene signature score in GC (B) and ITGB1 (C) -low and high group in TCGA database, respectively. D) Western blotting analysis demonstrated the expression of c-Jun in N008 cells upon following combinatorial rhGC_DEL_ treatment (50 ng/mL) with either vehicle control or pathway inhibitors, including FAK inhibitor (Defactinib, 5 μM), AKT inhibitor (LY294002, 20 μM), MAPK inhibitor (U0126, 20 μM), and YAP/TAZ (Verteporfin, 1 μM). E) Real-time qPCR analysis showed the marker of dedifferentiated Schwann cells expression in sh*ITGB1* N008 cells upon treatment with rhGC_DEL_ (n = 3 per group). F) Real-time qPCR analysis showed the marker of dedifferentiated Schwann cells expression in N008 cells upon treatment with anti-ITGB1 on the presence or absence of rhGC_DEL_ (n = 3 per group). In all panel, ***P* < 0.01, ****P* < 0.001, *****P* < 0.0001.


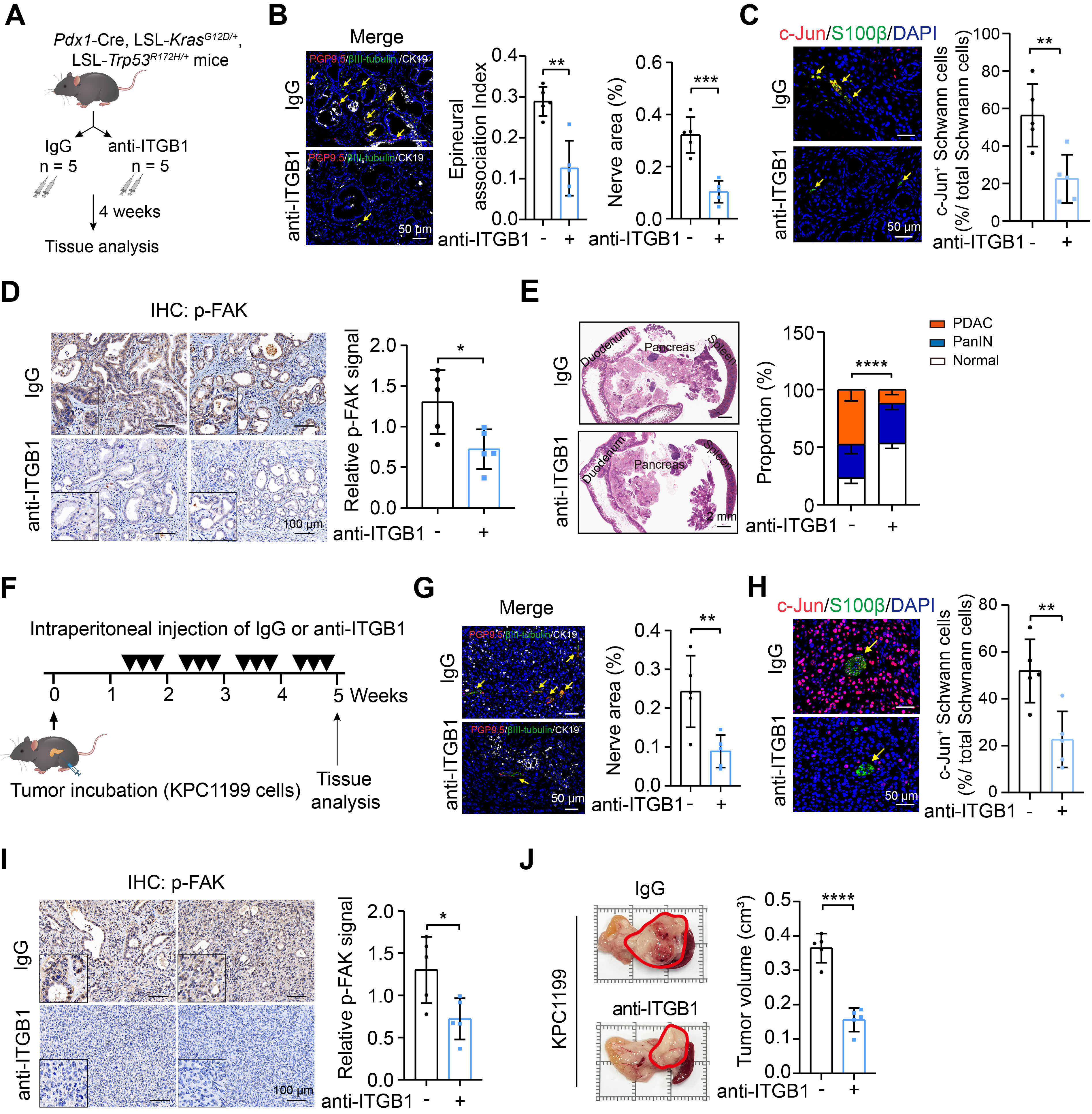


**Figure S7.** ITGB1 blockade disrupts cancer-nerve interaction and suppresses PDAC progression *in vivo*. A) Therapeutic schematic of intraperitoneal IgG versus anti-ITGB1 antibody administration in KPC mouse models (n = 5 per group). B) Representative imaging and quantitative analyses of ETAs frequency and nerve area in tumor tissues IgG- and anti-ITGB1-treated KPC mice. The yellow arrows indicated nerve. Scale bar, 50 μm. C) Immunofluorescence detection of c-Jun immunoreactivity in Schwann cells within tumor tissues upon IgG or anti-ITGB1 intervention. The yellow arrows indicated nerve. Scale bar, 50 μm. D) Phospho-FAK (Tyr397) expression profiles in tumor cells from IgG- versus anti-ITGB1-administered KPC tumor mice. Scale bar, 50 μm. E) Representative H&E-stained sections of pancreatic tumors with quantification of normal, pancreatic intraepithelial neoplasia (PanIN), and PDAC regions in IgG- and anti-ITGB1-treated KPC mice. Scale bar, 2 mm. F) Experimental timeline for intraperitoneal delivery of IgG or ITGB1-neutralizing antibodies in orthotopic KPC1199-derived PDAC models. G) Representative images and statistical evaluation of nerve area in pancreatic tumors from IgG and ITGB1 neutralizing antibody mice. The yellow arrows indicated nerve. Scale bar, 50 μm. H) The expression levels of c-Jun in Schwann cells within tumor tissues from IgG or ITGB1-neutralizing antibody administered orthotopic xenografts model. The yellow arrows indicated nerve. Scale bar, 50 μm. I) p-FAK immunohistochemical staining after IgG- and ITGB1-neutralizing antibody treatment in KPC1199 tumor. Scale bar, 100 μm. J) Macroscopic imaging (left) and quantification of orthotopic tumors volume in IgG- and ITGB1-neutralizing antibody-treated orthotopic xenografts model. In all panels, **P* < 0.05, *** P* < 0.01, ****P* < 0.001, *****P* < 0.0001.


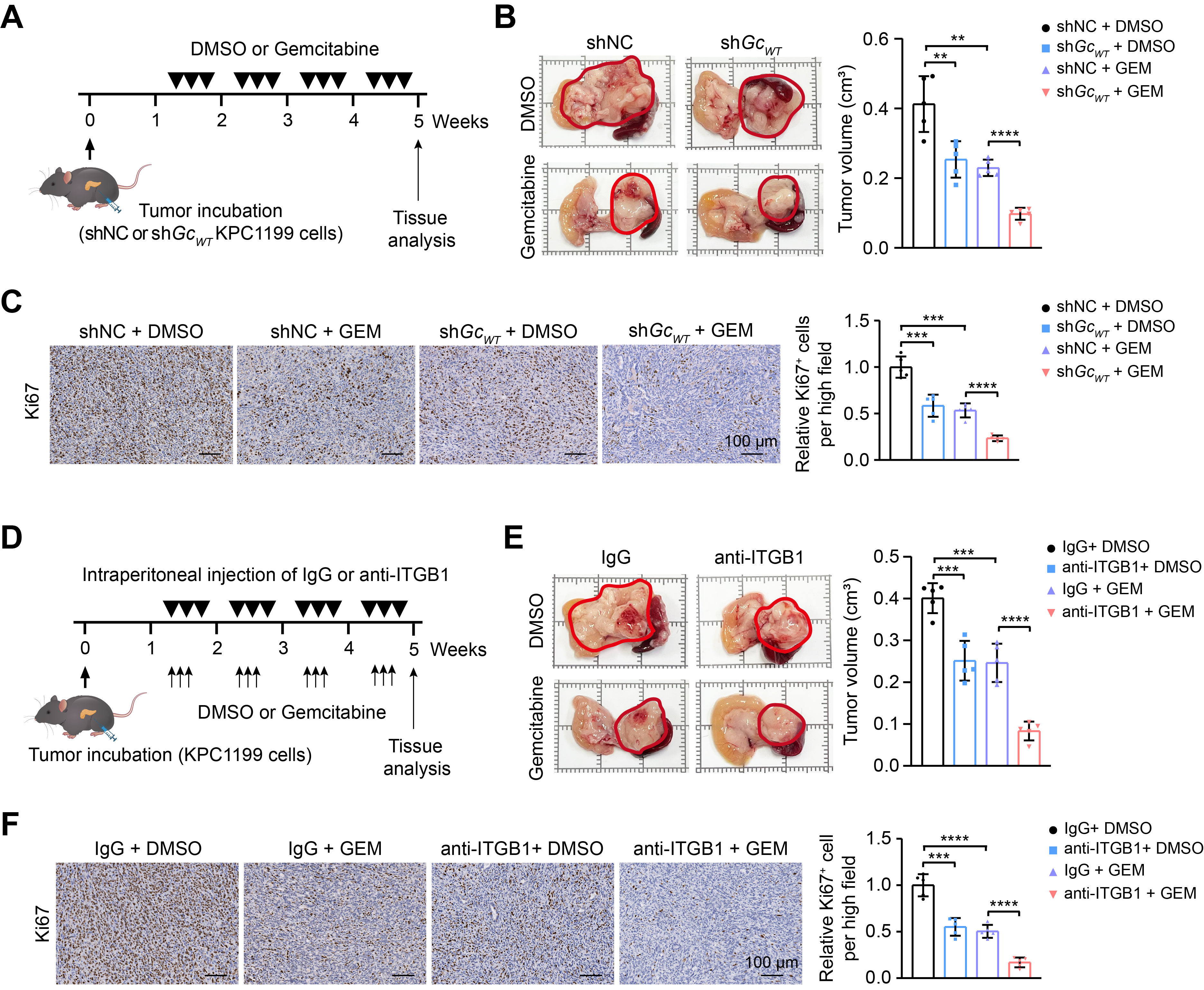


**Figure S8** GC-ITGB1 axis contributes to gemcitabine chemoresistance in pancreatic cancer. A) Schematic representation from gemcitabine (GEM, 30 mg/kg) or vehicle therapy in shNC or sh*Gc_WT_* KPC1199 derived orthotopic xenografts model (n = 5 per group). B) The volume of pancreatic tumor in mice between AAV-shNC and AAV- sh*Gc_WT_* plus gemcitabine or vehicle group was examined. C) Representative images of Ki67^+^ cells from pancreas tumor tissues of C57BL/6J mice treated with AAV-shNC and AAV-sh*Gc_WT_* combination with gemcitabine or vehicle. Scale bar: 100 μm. D) Treatment protocol showed intraperitoneal administration of IgG and anti-ITGB1 (5 mg/kg) combined with gemcitabine (30 mg/kg) or vehicle in orthotopic PDAC model (n = 5 per group). E) The volume changes of KPC119 tumor in mice received IgG/anti-ITGB1 combined with gemcitabine/vehicle treatment. F) Ki67 immunohistochemical staining after IgG/anti-ITGB1 plus gemcitabine/vehicle treatment in KPC1199 tumor-bearing mice. Scale bars, 100 μm. In all panels, *** P* < 0.01, ****P* < 0.001, *****P* < 0.0001.

**Supplementary Table 1.** The sequences for primers used in this study.

| **Gene** | **Forward primer (5’-3’)** | **Reverse primer (5’-3’)** |
| --- | --- | --- |
| *SOX9* | AGCGAACGCACATCAAGAC | CTGTAGGCGATCTGTTGGGG |
| *c-Jun* | TCCAAGTGCCGAAAAAGGAAG | CGAGTTCTGAGCTTTCAAGGT |
| *GFAP* | CTGCGGCTCGATCAACTCA | TCCAGCGACTCAATCTTCCTC |
| *SOX10* | CCATGTCAGATGGGAACCCC | CCGAAGTCGATGTGAGGCTT |
| *NCAM1* | GGCATTTACAAGTGTGTGGTTAC | TTGGCGCATTCTTGAACATGA |
| *L1CAM* | TGTCATCACGGAACAGTCTCC | CTGGCAAAGCAGCGGTAGAT |
| *MKi67* | AGAAGAAGTGGTGCTTCGGAA | AGTTTGCGTGGCCTGTACTAA |
| *PCNA* | ACACTAAGGGCCGAAGATAACG | ACAGCATCTCCAATATGGCTGA |
| *18S* | TGCGAGTACTCAACACCAACA | GCATATCTTCGGCCCACA |

**Supplementary Table 2.** The sequences for the overexpression vectors utilized in this study

GC (p.35_49del, rescue)

atgaagagggtcctggtactactgcttgctgtggcatttggacatgctttagagagaggccgggattatgaaaagaataaagtctgcaaggaattctcccatagaaaatttcccagtggcacgtttgaacaggtcagccaacttgtgaaggaagttgtctccttgaccgaagcctgctgtgcggaaggggctgaccctgactgctatgacaccaggacctcagcactgtctgccaagtcctgtgaaagtaattctccattccccgttcacccaggcactgctgagtgctgcaccaaagagggcctggaacgaaagctctgcatggctgctctgaaacaccagccacaggaattccctacctacgtggaacccacaaatgatgaaatctgtgaggcgttcaggaaagatccaaaggaatatgctaatcaatttatgtgggaatattccactaattacggacaagctcctctgtcacttttagtcagttacaccaagagttatctttctatggtagggtcctgctgtacctctgcaagcccaactgtatgctttttgaaagagagactccagcttaaacatttatcacttctcaccactctgtcaaatagagtctgctcacaatatgctgcttatggggagaagaaatcaaggctcagcaatctcataaagttagcccaaaaagtgcctactgctgatctggaggatgttttgccactagctgaagatattactaacatcctctccaaatgctgtgagtctgcctctgaagattgcatggccaaagagctgcctgaacacacagtaaaactctgtgacaatttatccacaaagaattctaagtttgaagactgttgtcaagaaaaaacagccatggacgtttttgtgtgcacttacttcatgccagctgcccaactccccgagcttccagatgtagagttgcccacaaacaaagatgtgtgtgatccaggaaacaccaaagtcatggataagtatacatttgaactaagcagaaggactcatcttccggaagtattcctcagtaaggtacttgagccaaccctaaaaagccttggtgaatgctgtgatgttgaagactcaactacctgttttaatgcCaaAggAccCctGctGaaAaaggaactatcttctttcattgacaagggacaagaactatgtgcagattattcagaaaatacatttactgagtacaagaaaaaactggcagagcgactaaaagcaaaattgcctgatgccacacccacggaactggcaaagctggttaacaagcactcagactttgcctccaactgctgttccataaactcacctcctctttactgtgattcagagattgatgctgaattgaagaatatcctg

GC (rescue)

atgaagagggtcctggtactactgcttgctgtggcatttggacatgctttagagagaggccgggattatgaaaagaataaagtctgcaaggaattctcccatctgggaaaggaggacttcacatctctgtcactagtcctgtacagtagaaaatttcccagtggcacgtttgaacaggtcagccaacttgtgaaggaagttgtctccttgaccgaagcctgctgtgcggaaggggctgaccctgactgctatgacaccaggacctcagcactgtctgccaagtcctgtgaaagtaattctccattccccgttcacccaggcactgctgagtgctgcaccaaagagggcctggaacgaaagctctgcatggctgctctgaaacaccagccacaggaattccctacctacgtggaacccacaaatgatgaaatctgtgaggcgttcaggaaagatccaaaggaatatgctaatcaatttatgtgggaatattccactaattacggacaagctcctctgtcacttttagtcagttacaccaagagttatctttctatggtagggtcctgctgtacctctgcaagcccaactgtatgctttttgaaagagagactccagcttaaacatttatcacttctcaccactctgtcaaatagagtctgctcacaatatgctgcttatggggagaagaaatcaaggctcagcaatctcataaagttagcccaaaaagtgcctactgctgatctggaggatgttttgccactagctgaagatattactaacatcctctccaaatgctgtgagtctgcctctgaagattgcatggccaaagagctgcctgaacacacagtaaaactctgtgacaatttatccacaaagaattctaagtttgaagactgttgtcaagaaaaaacagccatggacgtttttgtgtgcacttacttcatgccagctgcccaactccccgagcttccagatgtagagttgcccacaaacaaagatgtgtgtgatccaggaaacaccaaagtcatggataagtatacatttgaactaagcagaaggactcatcttccggaagtattcctcagtaaggtacttgagccaaccctaaaaagccttggtgaatgctgtgatgttgaagactcaactacctgttttaatgcCaaAggAccCctGctGaaAaaggaactatcttctttcattgacaagggacaagaactatgtgcagattattcagaaaatacatttactgagtacaagaaaaaactggcagagcgactaaaagcaaaattgcctgatgccacacccacggaactggcaaagctggttaacaagcactcagactttgcctccaactgctgttccataaactcacctcctctttactgtgattcagagattgatgctgaattgaagaatatcctg
